# BRAF inhibition protects against hearing loss in mice

**DOI:** 10.1126/sciadv.abd0561

**Published:** 2020-12-02

**Authors:** Matthew A. Ingersoll, Emma A. Malloy, Lauryn E. Caster, Eva M. Holland, Zhenhang Xu, Marisa Zallocchi, Duane Currier, Huizhan Liu, David Z.Z. He, Jaeki Min, Taosheng Chen, Jian Zuo, Tal Teitz

**Affiliations:** 1 Department of Pharmacology and Neuroscience, School of Medicine, Creighton University, Omaha, NE 68178, USA.; 2 Department of Biomedical Sciences, School of Medicine, Creighton University, Omaha, NE 68178, USA.; 3 Department of Otolaryngology, Xiangya Hospital, Central South University, Changsha 410008, China.; 4 Department of Chemical Biology and Therapeutics, St. Jude Children’s Research Hospital, Memphis, TN 38105, USA.

## Abstract

Hearing loss caused by noise, aging, antibiotics, and chemotherapy affects 10% of the world population, yet there are no Food and Drug Administration (FDA)-approved drugs to prevent it. Here, we screened 162 small-molecule kinase-specific inhibitors for reduction of cisplatin toxicity in an inner ear cell line and identified dabrafenib (TAFINLAR), a BRAF kinase inhibitor FDA-approved for cancer treatment. Dabrafenib and six additional kinase inhibitors in the BRAF/MEK/ERK cellular pathway mitigated cisplatin-induced hair cell death in the cell line and mouse cochlear explants. In adult mice, oral delivery of dabrafenib repressed ERK phosphorylation in cochlear cells, and protected from cisplatin- and noise-induced hearing loss. Full protection was achieved in mice with co-treatment with oral AZD5438, a CDK2 kinase inhibitor. Our study explores a previously unidentified cellular pathway and molecular target BRAF kinase for otoprotection and may advance dabrafenib into clinics to benefit patients with cisplatin- and noise-induced ototoxicity.

## Teaser

Dabrafenib, an FDA-approved oral anticancer drug, prevents cisplatin- and noise-induced hearing loss in animal models.

## INTRODUCTION

Seven hundred million people worldwide suffer from varying degrees of hearing loss (*1*, *2*). Cisplatin chemotherapy results in hearing loss in 40 to 60% of patients with cancer and impedes the development of language skills in young individuals (*3*–*5*). In modern society, noise-induced hearing loss is becoming more and more prominent because of ever-increasing levels of noise exposure. Despite extensive research efforts, to date, there are no Food and Drug Administration (FDA)–approved drugs to protect from noise-, cisplatin-, antibiotic-, or age-related hearing loss. Most candidate compounds currently in preclinical and clinical trials are related to antioxidants, vitamins, inflammation, and glutathione metabolism, such as sodium thiosulfate (STS), *N*-acetylcysteine, d-methionine, and dexamethasone (*1*, *2*, *6*). For example, STS, a direct inactivator of cisplatin and an antioxidant, was successful at lowering incidence of hearing loss by 48% in children with localized standard-risk hepatoblastoma when administered 6 hours after cisplatin chemotherapy, but lowered survival rate of treated patients with disseminated tumors (*7*–*9*). The steroid dexamethasone has been shown to be only partially effective in treating noise ototoxicity (*10*, *11*).

We initiated this work by developing an unbiased, phenotypic high-throughput screen (HTS) to identify small molecules that confer protection against cisplatin toxicity (*12*, *13*). Protein kinases are particularly attractive therapeutic targets as they regulate critical cellular functions, and many kinase inhibitors have been approved by the FDA for human treatment and, therefore, have great potential as therapeutics for hearing loss. Protein kinase phosphorylation cascades regulate major signal transduction pathways in all cell types in the body. Thus, inhibition of these pathways is likely to influence critical signal transduction pathways in the inner ear as well. Using an immortalized cell line derived from mouse cochleae and cultured in slow growth conditions that mimic nonmitotic cells growth (HEI-OC1 without γ-interferon) (*14*), we screened 162 kinase inhibitors in dose-response mode and identified a top-hit dabrafenib (TAFINLAR). Dabrafenib is a potent selective small-molecule inhibitor of BRAF kinase that is orally bioavailable and FDA-approved for treating multiple cancers with activated BRAF (BRAF V600E or V600K mutations) such as melanoma, small cell lung carcinoma, thyroid, and biliary tract cancers (*15*–*18*).

BRAF is a member of the Raf family of serine/threonine protein kinases. It plays an important role in the RAS/RAF/MEK [mitogen-activated protein kinase (MAPK) kinase]/ERK (extracellular signal–regulated kinase) signal transduction pathway (also known as part of the MAPK signaling cascade) and is involved in cell proliferation and cell survival (*19*). This pathway is deregulated in approximately one-third of all human cancers (*20*, *21*). Phosphorylation of BRAF activates the MEK protein, which, in turn, activates the downstream ERK protein kinase that phosphorylates a variety of substrates including nuclear transcription factors (*22*, *23*). BRAF is detected in the mouse inner ear starting at embryonic day 18.5 (E18.5) and is detected in postmitotic cochlear hair cells (HCs) and supporting cells (SCs) (*24*, *25*). It has been reported previously that phospho-ERK expression is up-regulated in the mouse inner ear upon mechanical and noise damage (*26*–*28*) and may be the cellular signal that senses cell damage and triggers cell death. Thus, inhibition of BRAF against ototoxic insults could reveal novel roles of this signaling pathway in postmitotic cochlear cells.

Repurposing FDA-approved drugs has recently become a particularly attractive and effective alternative in drug development, and several anticancer drugs are used for new medical indications (*29*). Repurposed drugs have shorter developmental times (5 to 8 years) and cost up to 40% less to bring to market than new chemical entities (10 to 15 years and >$2 billion) (https://ncats.nih.gov/preclinical/repurpose). On the basis of the wealth of information of dabrafenib in human use, it can be rapidly advanced to clinical trials in humans for hearing protection and treatment. Dabrafenib has several clear advantages as an otoprotective therapeutic candidate: (i) Dabrafenib is an FDA-approved drug delivered orally, unquestionably the most convenient route to administer drugs in the general population, and already has been approved for use by oral administration in humans in doses that are in the range we can test here for hearing protection. (ii) Dabrafenib penetrates the blood-brain barrier (*30*, *31*), which is similar to the blood-labyrinth barrier (*32*). This unique property to cross the blood-labyrinth barrier overcomes one of the major blockades of drug development for hearing loss. (iii) Dabrafenib has been developed as an anticancer drug (*16*–*18*) and is not likely an antioxidant compound per se and, thus, has the potential to not interfere but even synergize with cisplatin tumor-killing efficacy in some tumor types. (iv) Dabrafenib affects a new biological target, the BRAF kinase pathway, in the field of hearing protection and treatment. Given the large number of studies on specific and potent inhibitors of the BRAF/MEK/ERK pathway as anticancer drugs, combination therapy of multiple kinase inhibitors in this and additional pathways can offer even better protection at lower, less toxic doses. Dabrafenib (BRAF inhibitor) and trametinib (MEK inhibitor) combination therapy is used currently for melanoma and non–small lung carcinoma treatment and is more efficient than dabrafenib alone in inhibiting the pathway (*18*, *33*). Previously, we have identified CDK2 inhibitors as candidate therapeutics for hearing loss (*13*, *34*). Thus, the combination of dabrafenib and AZD5438 (a CDK2 inhibitor) may offer better efficacy against ototoxicity and lower general toxicity.

Here, we first validated dabrafenib and three additional BRAF inhibitors, two MEK1/2 inhibitors, and an ERK1/2 specific inhibitor in cisplatin-induced toxicity in mouse cochlear explants and tested the efficacy of dabrafenib alone or dabrafenib combined with AZD5438 to protect against cisplatin- and noise-induced hearing loss in adult mouse models when delivered orally. Our results demonstrate the key roles of the BRAF kinase and the BRAF/MEK/ERK and CDK2 pathways in stress activation and induction of apoptosis in postmitotic cochlear cells when evoked by both cisplatin and noise insults. This early phosphorylation cascade was activated in the cochlear SCs and was attenuated by small-molecule inhibitors of BRAF/MEK/ERK kinases. Our studies identify candidate therapeutics for hearing loss and highlight the key roles of BRAF/MEK/ERK and CDK2 pathways in postmitotic cochlear cells.

## RESULTS

### Dabrafenib is a top hit in a small-molecule kinase inhibitor screen

**Fig. 1:**
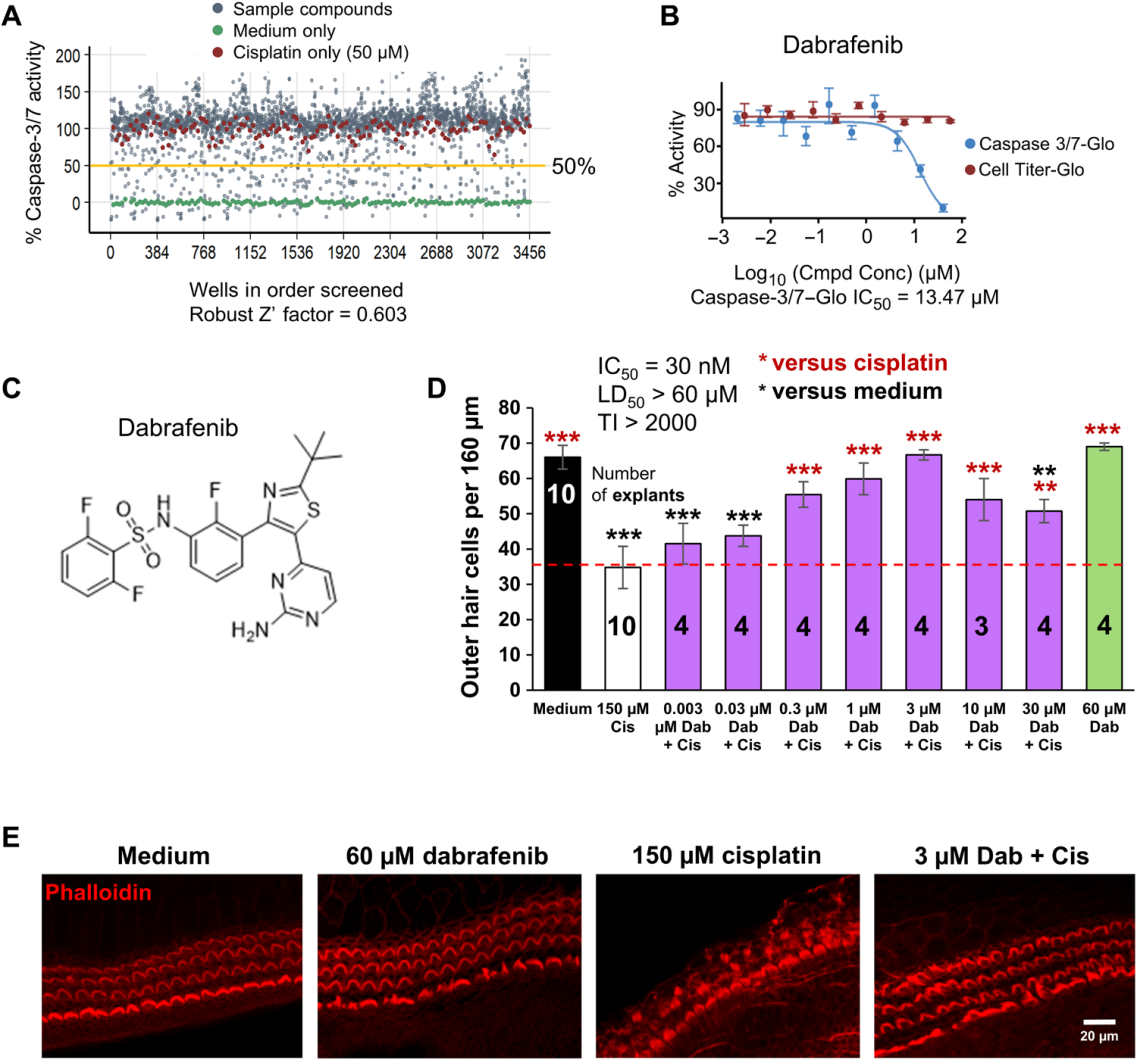
Dabrafenib protects from cisplatin-induced cell death in cell line and cochlear explant models. (**A**) Cell-based small-molecule screen in dose-response mode using HEI-OC1 cell line; hits were defined as compounds that reduce caspase-3/7 activity by 50% or more in the presence of 50 μM cisplatin. Medium alone (green dot), cisplatin alone (red dot), and compound + cisplatin (gray dot). (**B**) Dabrafenib dose-response curve from caspase-3/7 assay (A) in the presence of 50 μM cisplatin (blue), and Cell Titer-Glo dose-response curve of dabrafenib alone in HEI-OC1 cells (red). (**C**) Molecular structure of dabrafenib. (**D**) Dose-response of dabrafenib (Dab) in P3 FVB mouse cochlear explants treated with or without cisplatin. Medium alone (black), cisplatin alone (white), Dab alone (green), or Dab added 1 hour before cisplatin (150 μM) to P3 FVB cochlear explants treated for 24 hours (purple). Numbers inside each bar indicate number of explants counted per treatment. Number of outer hair cells (OHCs) per 160 μm of middle turn regions of the cochlea were counted by phalloidin staining, means ± SEM, **P < 0.01, ***P < 0.001 compared to cisplatin alone (red) and medium alone (black) by one-way analysis of variance (ANOVA) with Bonferroni post hoc test. (**E**) Representative confocal images of phalloidin-stained whole-mount middle turn cochlear explants treated with medium alone, 60 μM Dab, 150 μM cisplatin, or 3 μM Dab and 150 μM cisplatin for 24 hours are shown.

An unbiased screen for compounds protective against cisplatin ototoxicity (*12*, *13*) was conducted using the immortalized inner ear cell line HEI-OC1 derived from mouse cochlea (*14*). One hundred sixty-two unique small-molecule kinase-specific inhibitors were screened in dose-response mode for protection from 50 μM cisplatin. A caspase-3/7 activity assay was selected to measure apoptosis using a protocol that quantifies a luminescent product derived from the cleavage of caspase-3/7 substrate (Caspase-Glo 3/7 reagent). One hundred percent luminescence was defined by cells treated with cisplatin alone, and 0% was defined by untreated cells (Fig. 1A, fig. S1, table S1, and dataset in the Supplementary Materials).

All compounds were further characterized via Cell Titer-Glo cell viability assay to determine toxicity of the compound alone in HEI-OC1 cells (table S1 and dataset in the Supplementary Materials). The top hits include four BRAF-specific inhibitors: dabrafenib mesylate, vemurafenib, PLX-4720, and RAF-265. Of the compounds tested, dabrafenib was selected for further characterization because it is orally bioavailable, FDA-approved for treatment of metastatic melanoma and anaplastic thyroid cancer, and EU-approved for non–small cell lung carcinoma and because it can cross the blood-brain barrier (*15*–*18*, *35*, *36*). Dabrafenib demonstrated protection from cisplatin-induced cell death with a median inhibitory concentration (IC_50_) of 13.47 μM in the caspase-3/7 assay and was nontoxic to HEI-OC1 cells at all concentrations tested in the cell viability assay (Fig. 1, B and C). The other BRAF inhibitors had similar IC_50_ in the micromolar range and were nontoxic to cells (table S1).

### Dabrafenib and other MAPK inhibitors protect from cisplatin-induced HC death in mouse cochlear explants

Mouse cochlear explants are widely recognized as an alternative to in vivo cochlear models and pragmatic for focused drug screening (*37*). We next characterized dabrafenib’s protective effect using P3-P4 FVB mouse cochlear explants pretreated with dabrafenib for 1 hour, followed by 24-hour 150 μM cisplatin treatment, and counted the number of surviving outer HCs (OHCs) per 160 μm. Medium-alone, drug-alone, and cisplatin-alone treatments were used as controls. Dose-response experiments revealed that dabrafenib had a protective effect with an IC_50_ of 30 nM, a median lethal dose (LD_50_) of >60 μM, and a therapeutic index (TI), defined as LD_50_/IC_50_, of >2000 (Fig. 1, D and E; see the “Cochlear explants” section in Materials and Methods for comparison of IC_50_s in explants and cell line).

**Fig. 2:**
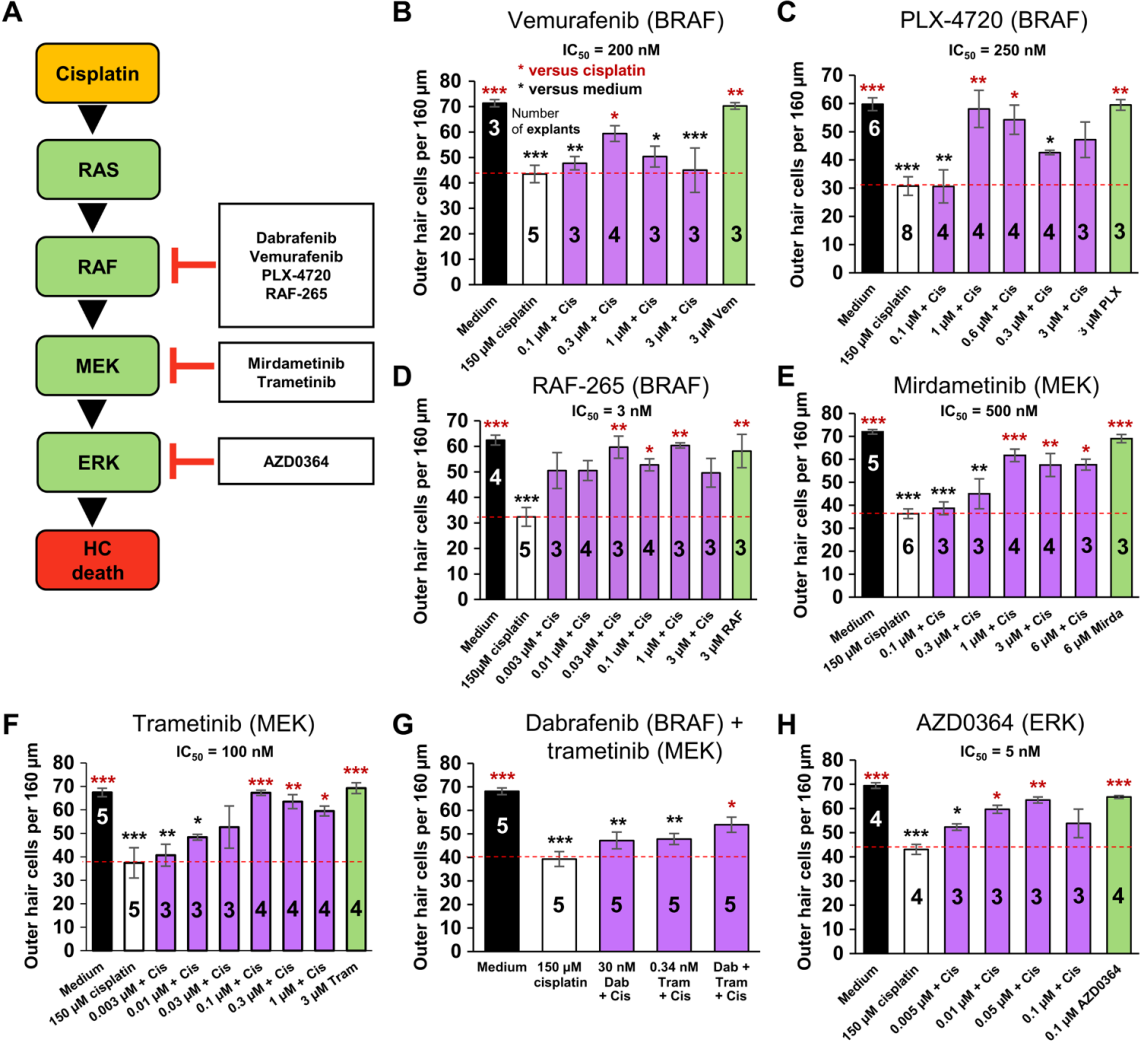
Additional top-hit BRAF and MAPK inhibitors protect against cisplatin ototoxicity in explant. (**A**) The putative BRAF/MEK/ERK cellular pathway and small-molecule protein kinase inhibitors in this pathway that were top hits in our inner ear cell line screen for protection from cisplatin-induced cell death. BRAF inhibitors vemurafenib (**B**), PLX-4720 (**C**), and RAF-265 (**D**); MEK1/2 inhibitors mirdametinib (**E**), trametinib (**F**), and combined dabrafenib and trametinib (**G**); and ERK1/2 inhibitor AZD-0364 (**H**) tested in the cochlear explant culture assay for protection against cisplatin-induced OHC death. Medium alone (black), cisplatin alone (white), compound alone (green), or compound added 1 hour before cisplatin (150 μM) to P3 FVB cochlear explants treated for 24 hours (purple). Numbers inside each bar indicate number of explants counted per treatment. Number of OHCs per 160 μm of middle turn regions of the cochlea were counted by phalloidin staining, means ± SEM, *P < 0.05, **P < 0.01, ***P < 0.001 compared to cisplatin alone (red) and medium alone (black) by one-way ANOVA with Bonferroni post hoc test.

Next, we sought to further verify BRAF and the MAPK signaling cascade as our target by testing additional top-hit kinase-specific inhibitors for protection from cisplatin-induced cell death in cochlear explants. In the canonical MAPK cascade, BRAF directly phosphorylates and activates MEK, which, in turn, phosphorylates and activates ERK (Fig. 2A) (*38*). Three other BRAF inhibitors vemurafenib (*39*), PLX-4720 (*40*), and RAF-265 (*41*, *42*) significantly mitigated cisplatin-induced OHC loss with IC_50_/TI’s of 200 nM/15.0, 250 nM/12.0, and 3 nM/666.7, respectively (Fig. 2, B to D). Two additional MEK inhibitors, trametinib (*43*) and mirdametinib (*44*), and an ERK specific inhibitor, AZD0364 (*45*), conferred significant protection from cisplatin with IC_50_/TI’s of 100 nM/40.0, 500 nM/6.0, and 5 nM/200.0, respectively (Fig. 2, E to H). Moreover, an FDA-approved combination of dabrafenib and trametinib administered in a molar ratio of 1:80 is routinely used for treatment of BRAF-mutated cancers as it is demonstrated to effectively enhance the inhibition of the BRAF pathway and increase survival of patients (*18*, *33*). We tested this combination in our explant model at suboptimal concentrations in which neither drug alone had significant protective effect, but in which the compounds combined had significant protection against cisplatin-induced OHC loss (Fig. 2G). Confocal images of phalloidin-stained tissues for all compounds are shown in fig. S2. Note that for all compounds tested with cisplatin treatment in cochlear explants, we observe a Gaussian curve: At low concentrations, there is no protection, and at high concentrations, the compound in combination with cisplatin is toxic to the HCs. Together, these data further validate BRAF and its downstream effectors as therapeutic targets for protection from cisplatin-induced ototoxicity. Dabrafenib outperformed all compounds tested in terms of IC_50_ (30 nM; Fig. 1D), except RAF-265 and AZD0364, which were even more potent, but because of dabrafenib’s FDA status, best TI (>2000), and permeability through the blood-brain barrier, we continued with this compound for in vivo testing.

Moreover, to benchmark dabrafenib against other drugs currently involved in clinical trials, we compared its IC_50_ and TI to those of compounds previously reported using an identical P3 FVB explant model. Included are kenpaullone, STS, ebselen, d-methionine, and dexamethasone, which have IC_50_/TI’s of 0.2 μM/150, 2.1 μM/285, 10.8 μM/1.4, 98.4 μM/1.0, and >0.25 μM/20, respectively (*13*, *46*). Comparatively, dabrafenib is more potent and has greater TI than all other compounds, indicating that it is an excellent candidate for further in vivo testing in adult mice.

### Dabrafenib blocks cisplatin induction of MAPK phosphorylation cascade

**Fig. 3:**
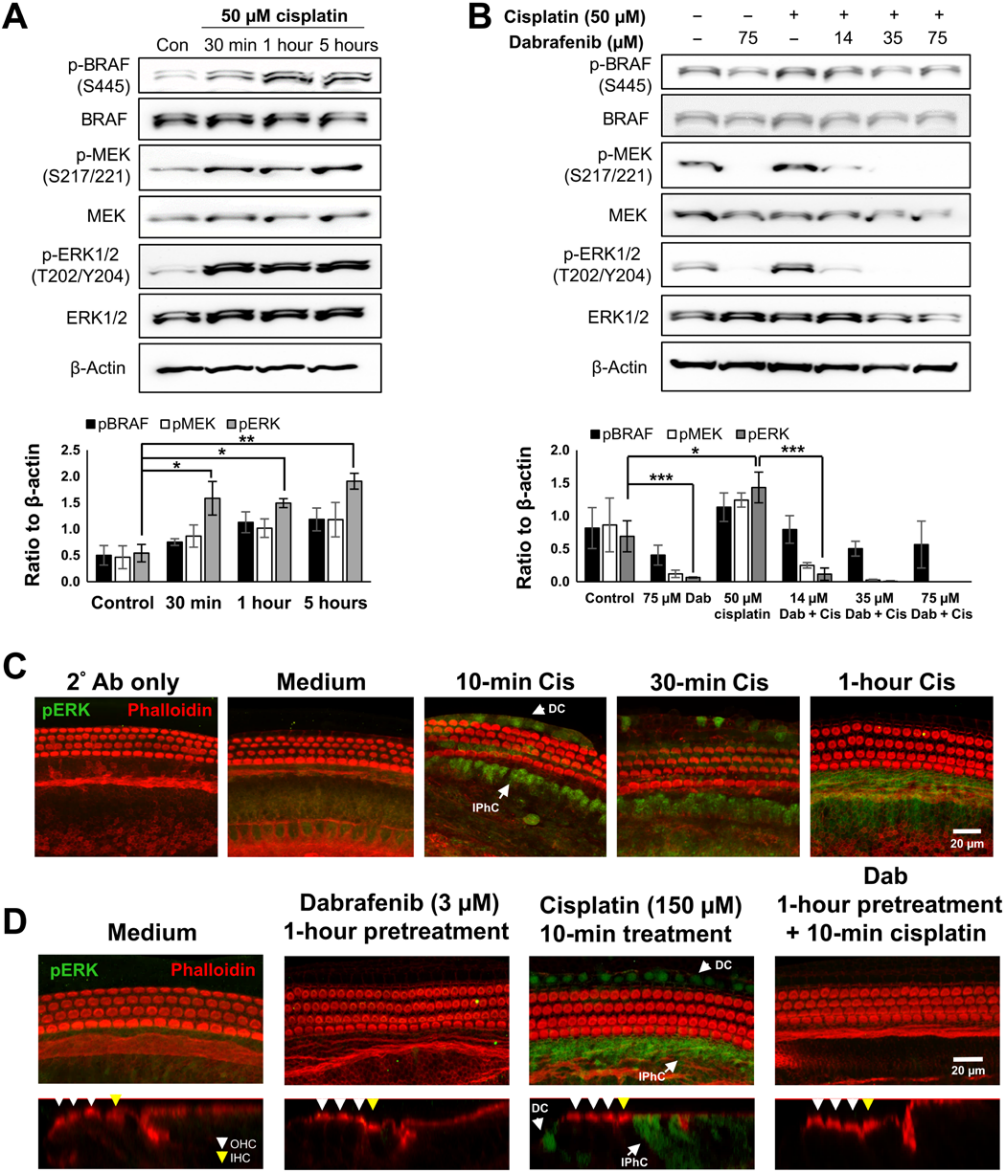
Dabrafenib mitigates cisplatin-activated BRAF signaling cascade. (**A**) Representative Western blot images of BRAF, ERK, and MEK phosphorylation in HEI-OC1 cells upon 50 μM cisplatin treatment for 30 min, 1 hour, and 5 hours. Phosphorylated protein bands were normalized to β-actin and averaged, means ± SEM, *P < 0.05, **P < 0.01 by one-way ANOVA with Bonferroni post hoc test. *n* = 4. (**B**) Representative Western blot images (*n* = 3) of BRAF, ERK, and MEK phosphorylation upon combined dabrafenib (14, 35, or 75 μM) and cisplatin (50 μM) treatment in HEI-OC1 cells. Cells are pretreated with dabrafenib for 1 hour before 1-hour cisplatin treatment. Medium alone, cisplatin alone, and 75 μM dabrafenib alone used as controls. Phosphorylated protein bands were normalized to β-actin and averaged, means ± SEM, *P < 0.05, ***P < 0.001 by one-way ANOVA with Bonferroni post hoc test. *n* = 3. (**C**) Representative phalloidin (red) and phosphorylated ERK (pERK) (green) stained confocal images of P3 FVB whole-mount middle turn mouse cochlea explants pretreated with 3 μM dabrafenib (Dab) for 1 hour before 10 min cisplatin (150 μM) exposure. Deiters’ cells (DC) and inner phalangeal cells (IPhC) with labeled arrows. *n* = 6 cochlea. (**D**) Representative phalloidin (red)– and pERK (green)–stained confocal images of P3 FVB whole-mount middle turn mouse cochlea explants pretreated with 3 μM dabrafenib (Dab) for 1 hour before 10 min cisplatin (150 μM) exposure. Ortho section shown below in which OHCs are identified with white arrows, inner HCs (IHCs) are identified with yellow arrows, and pERK-positive DCs and IPhCs are identified with labeled arrows. *n* = 6 cochlea.

Because dabrafenib is a potent inhibitor of cisplatin-induced cell death in HEI-OC1 cells and cochlear explants, we next demonstrated cisplatin activation of BRAF and its downstream targets in these models. HEI-OC1 cells were treated with 50 μM cisplatin, the same concentration used for the caspase-3/7 screen, for 30 min, 1 hour, and 5 hours, and whole-cell lysates were analyzed by Western blot. Cisplatin was found to increase phosphorylation, and thus activation, of BRAF, MEK, and ERK over time with significant increase in ERK phosphorylation at all three time points (Fig. 3A). Cells were then pretreated with increasing concentrations of dabrafenib for 1×, 2.5×, and 5× the IC_50_ in HEI-OC1 cells. Cells were treated with compound for 1 hour, followed by 50 μM cisplatin treatment for 1 hour to determine dabrafenib’s ability to block cisplatin-induced changes in signaling via Western blot. Dabrafenib treatment alone at the highest concentration tested was used as a control. While cisplatin again increased BRAF, MEK, and ERK phosphorylation compared with control cells, dabrafenib mitigated these changes in a dose-dependent manner (Fig. 3B).

Because significant changes in signaling were observed in ERK, P3-P4 FVB cochlear explants were treated with 150 μM cisplatin for 10 min, 30 min, and 1 hour, and then stained for phalloidin and phosphorylated ERK (pERK). Tissue samples were then imaged via confocal microscopy. Rapid phosphorylation of ERK was observed at 10 min, followed by decreasing signal at 30 min and 1 hour. Notably, pERK signal was observed initially in SCs, in particular Deiters’ (DC) and inner phalangeal cells (IPhC) regions, but not HCs, and propagated to surrounding cells (Fig. 3C). To determine whether dabrafenib prevents cisplatin-induced ERK activation, we pretreated cochlear explants with 3 μM dabrafenib for 1 hour, followed by 10 min cisplatin exposure. While untreated cochleae expressed low levels of pERK, cisplatin-induced ERK phosphorylation was observed again in DC and IPhC regions, while dabrafenib treatment prevented ERK activation (Fig. 3D). To verify whether ERK is activated after cisplatin treatment in the SCs and not in the HCs, we costained the explants with myosin VIIa that labels HCs only and showed there is no overlap between the cells that activate ERK and cells that stained positive with HC-specific marker (fig. S3). Combined, these data demonstrate that cisplatin is a potent inducer of the MAPK phosphorylation cascade, while dabrafenib mitigates cisplatin activation of the pathway.

### Dabrafenib protects against cisplatin-induced HC loss in zebrafish in vivo

Lateral line neuromasts of zebrafish are a well-established model for the study of drug protection from cisplatin or aminoglycoside toxicity because their HCs are considered homologous to mammalian HCs and readily accessible to compounds in vivo (*47*–*49*). As in our previous studies, MI1 (medial neuromast 1) and O1-2 (otic line) neuromasts were examined in *Tg(brn3c:GFP)* zebrafish larvae 5 days after fertilization (*50*, *51*). Larvae were pretreated with dabrafenib for 1 hour, followed by 6 hours with high-dose 400 μM cisplatin, with or without dabrafenib treatment, and allowed to recover for 1 hour in fresh water. Green fluorescent protein (GFP) (*brn3c*) and otoferlin staining were used to identify neuromast HCs. In fig. S4, larvae treated with cisplatin alone showed significant HC loss compared to control dimethyl sulfoxide (DMSO)–alone animals, while those cotreated with 100 nM dabrafenib had significant protection compared to cisplatin alone. Dabrafenib-alone treatment at all concentrations tested demonstrated no toxic effect. Thus, dabrafenib protects against cisplatin-induced HC death in zebrafish in vivo.

### Dabrafenib protects against cisplatin-induced hearing loss in adult mice in vivo

**Fig. 4:**
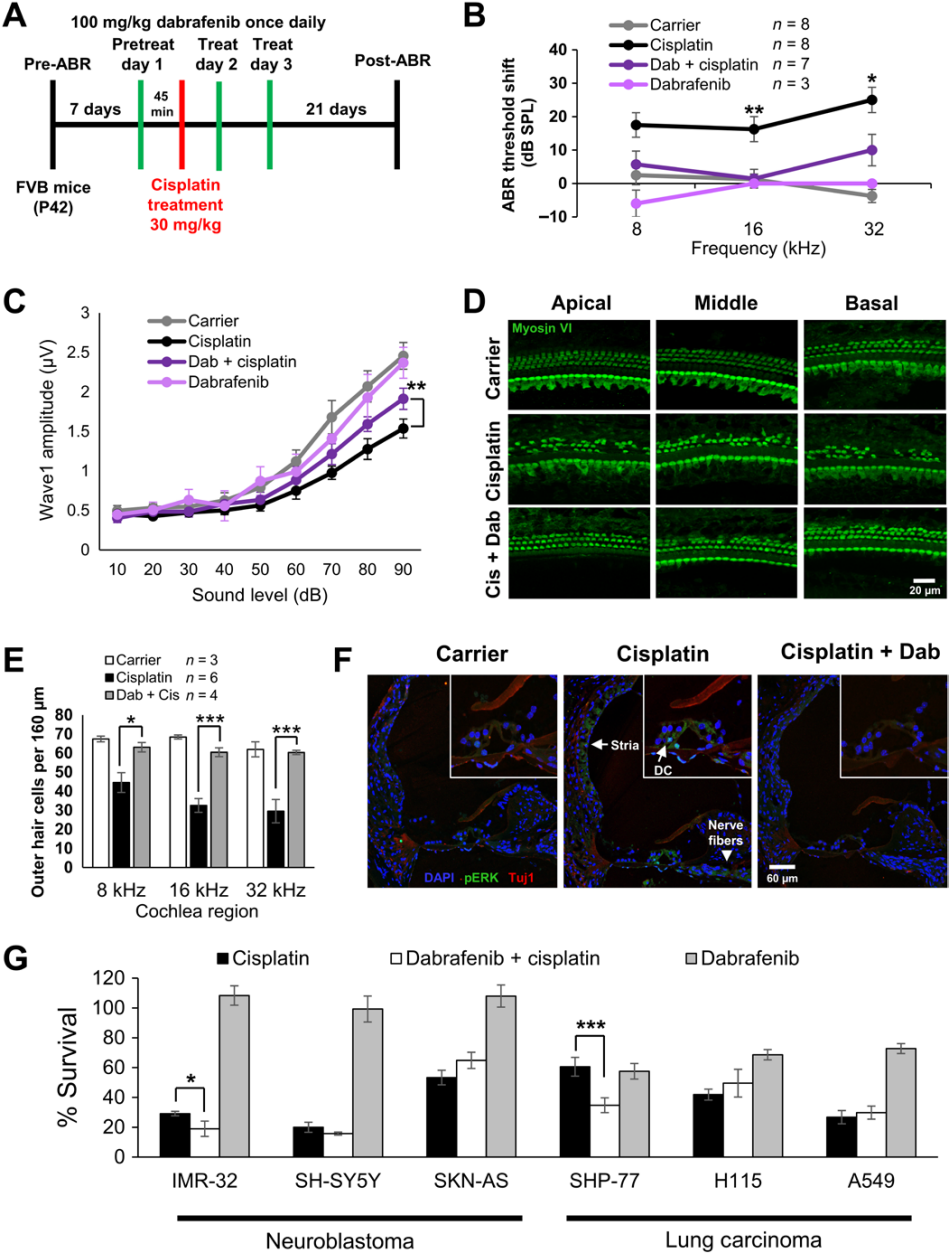
Dabrafenib protects against cisplatin-induced hearing loss in adult mice and does not inhibit cisplatin tumor-killing efficacy. (**A**) Schedule of administration of dabrafenib and cisplatin to adult P42 FVB mice. (**B**) ABR threshold shifts following protocol from (A). Untreated controls (gray), cisplatin alone (black), dabrafenib alone (light purple), and dabrafenib and cisplatin (dark purple). (**C**) Amplitudes of ABR wave 1 at 16 kHz from (B). (B and C) Means ± SEM, *P < 0.05, **P < 0.01, compared to cisplatin alone by two-way ANOVA with Bonferroni post hoc test. (**D**) Representative myosin VI–stained confocal images of 8-, 16-, and 32-kHz cochlear regions from carrier alone, cisplatin alone, and combined dabrafenib (Dab) and cisplatin-treated mice. (**E**) Number of OHCs per 160 μm in 8-, 16-, and 32-kHz cochlear regions were counted by myosin VI staining; data shown as means ± SEM, *P < 0.05, ***P < 0.001 by two-way ANOVA with Bonferroni post hoc test. (**F**) Representative 4′,6-diamidino-2-phenylindole (DAPI) (blue), pERK (green), and Tuj1 (red) immunofluorescence-stained cochlear sections from P42 FVB mice treated with carrier alone, 1-hour cisplatin (30 mg/kg), or dabrafenib (100 mg/kg) 45 min pretreatment followed by 1-hour cisplatin. Higher-magnification images of the organ of Corti region are inlaid in the upper right corner. *n* = 3 mice. (**G**) Cell Titer-Glo percent cell survival of neuroblastoma and lung carcinoma cell lines pretreated 1 hour with dabrafenib followed by 48-hour combined cisplatin and dabrafenib treatment. Data shown as means ± SEM, *P < 0.05, ***P < 0.001 compared to cisplatin alone by one-way ANOVA with Bonferroni post hoc test. *n* = 6.

Dabrafenib exhibited superior potency and TI in mouse cochlear explants when evaluated with other MAPK inhibitors in addition to previously examined compounds currently in clinical trials for cisplatin ototoxicity; thus, we next tested the drug in adult mice. We used oral gavage for dabrafenib delivery as this method is readily accessible in a clinical setting and in the general population. As described in Fig. 4A, baseline auditory brainstem response (ABR) thresholds for P42 FVB mice were established 1 week before experimental procedures. Mice were pretreated with dabrafenib (100 mg/kg), one-third of the highest reported daily nontoxic dose in mice and comparable to the daily dose approved for humans (*15*, *18*, *52*), 45 min before previously optimized cisplatin (30 mg/kg) intraperitoneal injection (*13*). Then, mice were given additional dabrafenib doses at 24 and 48 hours after cisplatin exposure. Animals were allowed 21 days after cisplatin injection to recover, and final ABR thresholds were recorded. Mice treated with carrier or dabrafenib alone showed no significant threshold shift or change in body weight. Cisplatin-treated animals had significantly elevated threshold shifts at 8-, 16-, and 32-kHz frequencies, consistent with previous studies (*13*, *34*). Animals cotreated with dabrafenib and cisplatin had significantly reduced threshold shifts, with average reduction of 14.9 dB at 16 and 32 kHz. Notably, dabrafenib provided nearly full protection at 16 kHz (Fig. 4B and fig. S8). Similarly, at D14, the wave 1 amplitudes of ABRs at 16 kHz with various sound stimulus intensities were also higher in dabrafenib and cisplatin cotreated mice than in cisplatin-alone–treated mice and significant at 90-dB sound pressure level (SPL) (Fig. 4C).

As shown in Fig. 4 (D and E), morphological analysis of cochlea revealed significant OHC loss in cisplatin-alone–treated animals compared to controls, with greatest loss observed in the 32-kHz region, which is consistent with ABR threshold shifts. Loss of OHCs was limited in animals treated with combined dabrafenib and cisplatin, demonstrating the drug’s protective effect. No OHC loss was observed in mice treated with dabrafenib alone. Last, ERK phosphorylation in the cochlear SCs was observed in adult FVB mice 1 hour after cisplatin intraperitoneal administration, and the phosphorylation was attenuated by dabrafenib pretreatment (Fig. 4F). ERK phosphorylation was also observed in the areas of stria vascularis in the inner ear and in the nerve fiber bundles innervating the HCs, and dabrafenib pretreatment in vivo down-regulated the phosphorylation (Fig. 4F). Hence, pERK immunostaining, ABR threshold measurements, and morphological OHC counts strongly indicate that oral delivery of dabrafenib prevents ERK phosphorylation in cochlear tissues after cisplatin treatment and has a protective effect against cisplatin-induced hearing loss in adult mice, while dabrafenib treatment alone has no toxic effect.

### Dabrafenib does not interfere with cisplatin tumor-killing efficacy

While dabrafenib is demonstrated to protect against ototoxicity in multiple models, it is also vital to ascertain whether the drug interferes with cisplatin’s tumor-killing efficacy before it can be considered for clinical use. In clinical settings, cisplatin is used to treat several tumor types, including neuroblastoma and lung carcinoma. Therefore, three human neuroblastoma cell lines, IMR-32, SH-SY5Y, and SK-N-AS, as well as three human lung carcinoma cell lines, SHP-77 small cell, H1155 non–small cell, and A549 non–small cell, were used to determine whether dabrafenib affected cisplatin’s tumor killing ability. Cells were pretreated with dabrafenib alone for 1 hour, followed by 15 μM cisplatin (200 μM for A549) plus 35 μM dabrafenib treatment (3× protective IC_50_ in HEI-OC1 cells) for 48 hours and then assayed via Cell Titer-Glo to measure viability. Medium-alone–, cisplatin-alone–, and dabrafenib-alone–treated cells were used as controls, and viability was reported as percent survival compared to medium alone (Fig. 4E). Dabrafenib was found to have no inhibitory effect on cisplatin-induced tumor cell death; in fact, dabrafenib increased tumor cell death when combined with cisplatin in IMR-32 and SHP-77 cell lines. Together, these data indicate that dabrafenib does not interfere with cisplatin tumor-killing efficacy and, in some tumors, works with cisplatin to increase tumor cell death.

### Dabrafenib mitigates noise-induced hearing loss in adult mice in vivo

**Fig. 5:**
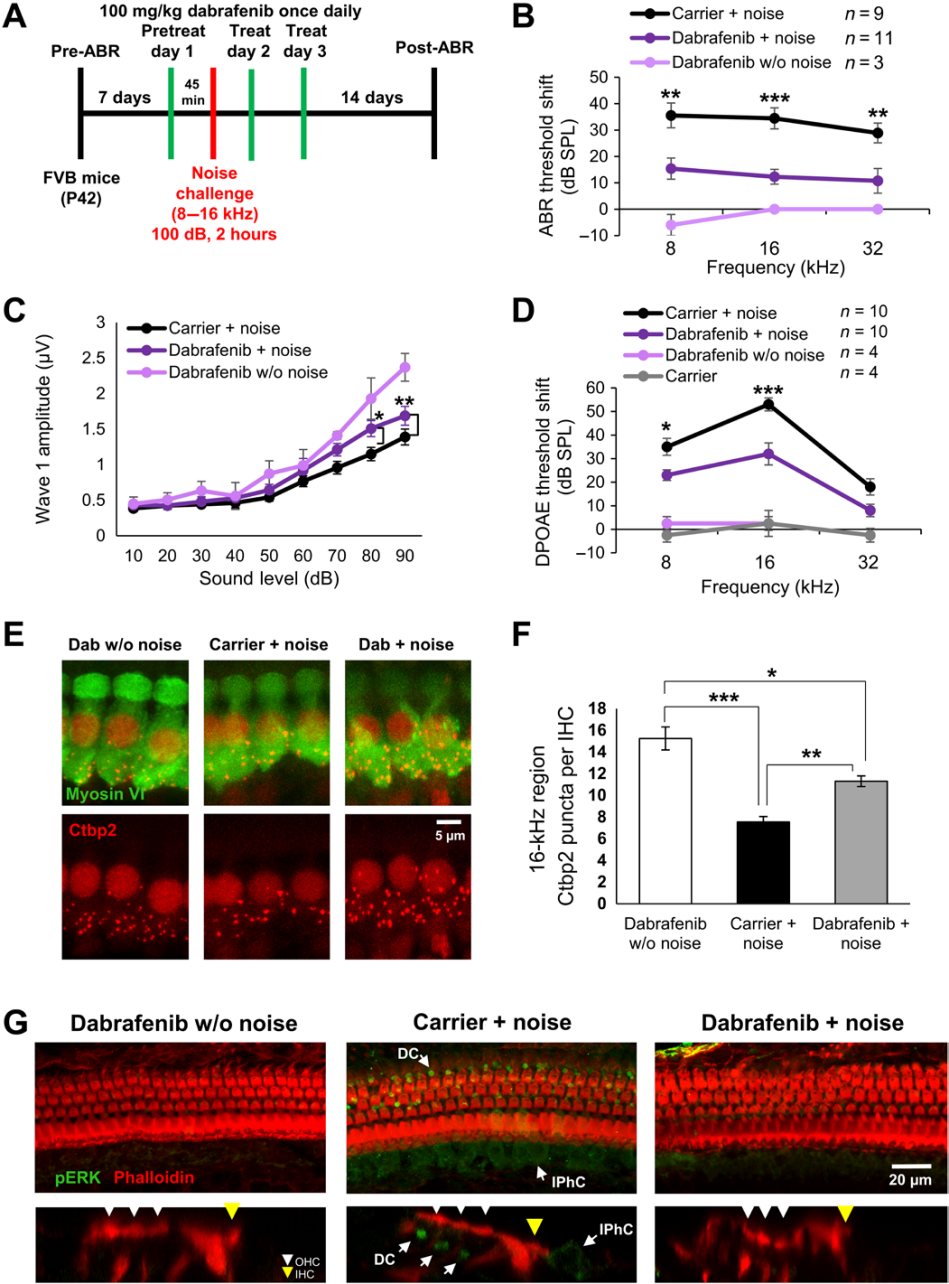
Dabrafenib pretreatment protects against noise-induced hearing loss in adult mice. (**A**) Schedule of administration of dabrafenib and noise exposure to adult P42 FVB mice. (**B**) ABR threshold shifts following protocol from (A). Dabrafenib treatment without noise exposure (light purple), noise alone (black), and dabrafenib treatment with noise exposure (dark purple). (**C**) Amplitudes of ABR wave 1 at 16 kHz from (B). (**D**) DPOAE threshold shifts following protocol from (A). Carrier without noise exposure (gray), dabrafenib treatment without noise exposure (light purple), noise alone (black), and dabrafenib treatment with noise exposure (dark purple). (B to D) Means ± SEM, *P < 0.05, **P < 0.01, ***P < 0.001 compared to noise alone by two-way ANOVA with Bonferroni post hoc test. (**E**) Representative Ctbp2 puncta (red)– and myosin VI (green)–stained confocal images of IHCs in whole-mount mouse cochlea at the 16-kHz region. (**F**) Number of Ctbp2 puncta per IHC at the 16-kHz cochlear region were counted; data shown as means ± SEM, *P < 0.05, **P < 0.01, ***P < 0.001 by one-way ANOVA with Bonferroni post hoc test. *n* = 4 cochlea. (**G**) Representative phalloidin (red) and pERK (green) immunofluorescence-stained adult FVB mice cochlea. Mice were exposed to noise following protocol from (A), then euthanized, and cochlea-fixed 3 hours after noise exposure. Ortho section below shows OHCs identified with white arrows, IHCs identified with yellow arrows, and pERK-positive DCs and IPhCs identified with labeled arrows. *n* = 2 cochlea.

Previous studies have revealed that compounds that provide protection from cisplatin-induced hearing loss may also protect against noise-induced hearing loss (*13*). To test whether dabrafenib protects from noise-induced hearing loss, we used a protocol previously published in which, 5 to 7 days after baseline ABR and DPOAE thresholds are established, mice are exposed to 100 dB of noise in the 8- to 16-kHz octave band for 2 hours, which results in permanent ABR and DPOAE threshold shifts of 30 to 50 dB observed 14 days after noise exposure (*13*). Experimental animals were given dabrafenib (100 mg/kg) via oral gavage 45 min before noise insult and two additional doses at 24 and 48 hours after noise exposure (Fig. 5A). Dabrafenib-treated animals had significantly lower ABR threshold shifts compared with carrier alone, with an average protection of 16.8 dB at the three frequencies tested (Fig. 5B and fig. S8). Dabrafenib-treated mice without noise exposure exhibited no significant threshold shift, change in body weight, or general behavior. In addition, while noise reduced 16-kHz ABR wave 1 amplitude, dabrafenib significantly mitigated this effect at 80- and 90-dB SPL (Fig. 5C). In a similar experiment, mice subjected to noise exposure exhibited a sharp increase in DPOAE threshold shifts at 8 and 16 kHz, while dabrafenib treatment provided significant protection (Fig. 5D).

Cochleae from these animals were collected and stained for myosin VI for morphological analysis. While no significant OHC loss was observed, previous studies report noise-injury results in dysfunction of synaptic ribbons where inner HCs (IHCs) form synaptic contacts with neuronal fibers (*53*, *54*). To this end, cochleae were costained for synaptic ribbon scaffolding protein, Ctbp2, to measure loss of synaptic ribbon integrity in IHCs at the 16-kHz region (Fig. 5, E and F). Compared to control mice treated with dabrafenib alone and no noise exposure, carrier-alone mice exposed to noise had significantly reduced numbers of Ctbp2-puncta per IHC. Treatment with dabrafenib partially rescued loss of puncta in mice and results correlate with ABR threshold shifts at 16 kHz. Furthermore, we sought to determine whether noise insult induced phosphorylation of ERK in cochleae, similar to that observed upon cisplatin insult. Staining mouse cochleae immediately and 3 hours after noise exposure for phalloidin and pERK revealed that noise activates ERK in SCs, in particular DCs and IPhCs (Fig. 5G and fig. S5), closely resembling images from cisplatin-treated cochleae (Figs. 3, C and D, and 4F). Dabrafenib pretreatment reduced ERK phosphorylation in these SCs (Fig. 5G). Therefore, combined ABR threshold shift data and Ctbp2-puncta measurements demonstrate that dabrafenib pretreatment provides significant protection from noise-induced ototoxicity in adult mice in vivo and blocks noise activation of the MAPK pathway.

### Combined dabrafenib and AZD5438 treatment prevents hearing loss after noise exposure in adult mice in vivo

**Fig. 6:**
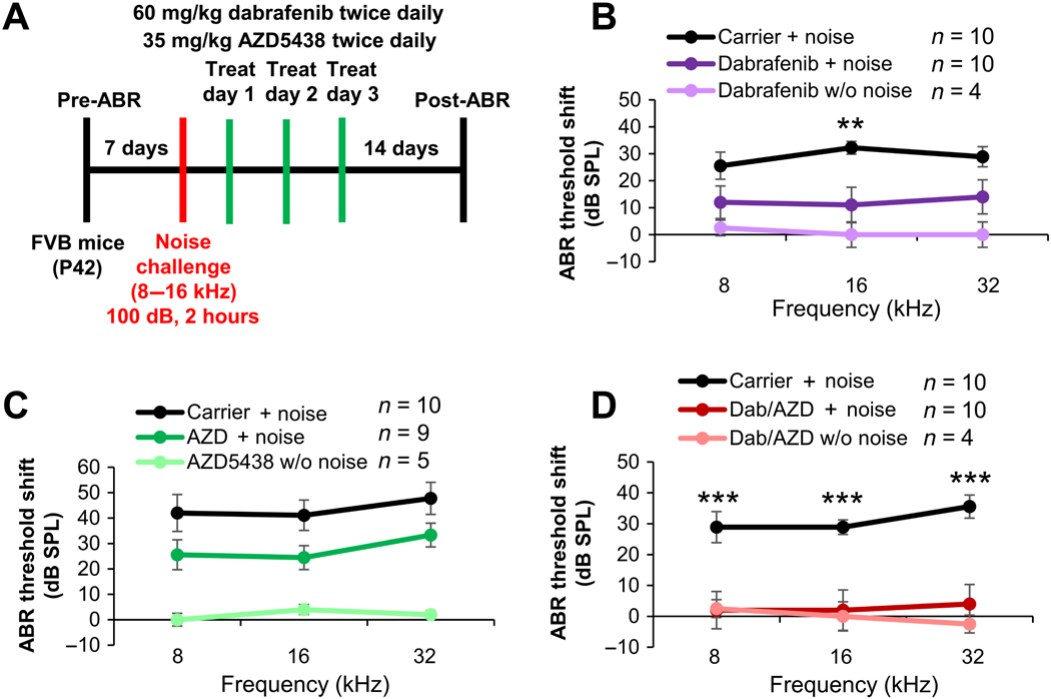
Dabrafenib and AZD5438 posttreatment protect against noise-mediated hearing loss in adult mice. (**A**) Schedule of administration of compound and noise exposure to adult P42 FVB mice. (**B**) ABR threshold shifts recorded 14 days after 100-dB 8- to 16-kHz octave band noise for 2 hours for control and dabrafenib (60 kg/mg) twice daily treated mice by oral gavage. Dabrafenib treatment without noise exposure (light purple), noise alone (black), and dabrafenib treatment with noise exposure (dark purple). Means ± SEM, **P < 0.01 compared to noise alone by two-way ANOVA with Bonferroni post hoc test. (**C**) ABR threshold shifts recorded 14 days after 100-dB 8- to 16-kHz octave band noise for 2 hours for control and AZD5438 (35 kg/mg) twice daily treated mice by oral gavage. AZD5438 treatment without noise exposure (light green), noise alone (black), and dabrafenib treatment with noise exposure (dark green). The values were not significant compared to noise alone by two-way ANOVA with Bonferroni post hoc test. (**D**) ABR threshold shifts recorded 14 days after 100-dB 8- to 16-kHz octave band noise for 2 hours for control and combined dabrafenib (60 kg/mg) and AZD5438 (35 mg/kg) twice daily treated mice by oral gavage. Dabrafenib and AZD5438 treatment without noise exposure (light red), noise alone (black), dabrafenib, and AZD5438 treatment (dark red). Means ± SEM, ***P < 0.001 compared to noise alone by two-way ANOVA with Bonferroni post hoc test.

Noise injury cannot always be predicted, and pretreatment is often not an option. Therefore, we sought to determine whether dabrafenib confers protection when administered after noise exposure. To this end, we modified our previous noise experimental protocol (Fig. 5A) to instead give mice dabrafenib treatment at 24, 48, and 72 hours after noise insult (Fig. 6A). Also, in an attempt to improve therapeutic effect, instead of giving mice one dose of drug at 100 mg/kg daily, we administered dabrafenib by oral gavage at 60 mg/kg twice daily, 8 hours between treatments, because the compound has a half-life of roughly 8 hours in circulation (*55*). Dabrafenib treatment in this regimen resulted in significantly reduced threshold shifts, with a mean reduction of 21.0 dB at frequencies of 16 kHz (Fig. 6B). Thus, the data indicate that dabrafenib confers protection from noise-induced hearing loss after noise exposure in adult mice in vivo.

Recently, we identified several CDK2 inhibitors that protect against cisplatin- and noise-induced hearing loss (*13*, *34*). AZD5438 is a second-generation CDK2 inhibitor that is orally bioavailable; thus, we sought to determine whether the compound could be combined with dabrafenib to provide enhanced protection from cisplatin- and noise-induced hearing loss (fig. S6A). Initially, we tested a combination of 30 nM dabrafenib and 0.34 nM AZD5438 in our cisplatin-exposed cochlear explant model and showed that it provided enhanced protection compared to each drug alone (fig. S6B). To ensure that AZD5438 and combined dabrafenib/AZD5438 treatment did not interfere with cisplatin tumor-killing efficacy, neuroblastoma and lung cell carcinoma cell lines were tested (fig. S6C). Apart from cell line SKN-AS for AZD5438 alone, AZD5438 alone and combined dabrafenib and AZD4538 treatments did not interfere with or enhanced cisplatin-mediated tumor cell death in all cell lines tested. In addition, mouse pretreatment experiments using a single dose of AZD5438 (75 mg/kg) delivered via oral gavage demonstrated that AZD5438 protected against both cisplatin- and noise-induced hearing loss (fig. S7, A to D). However, repeating the post-noise treatment experiment revealed that AZD5438 (35 mg/kg) delivered twice daily by oral gavage did not significantly reduce ABR threshold shifts in mice (Fig. 6C).

Last, we sought to determine whether combined dabrafenib and AZD5438 could provide enhanced protection after noise exposure. Compared to carrier-alone mice, mice treated with dabrafenib (60 mg/kg) and AZD5438 (35 mg/kg) twice daily 24, 48, and 72 hours after noise exposure exhibited significantly reduced ABR threshold shifts, with a mean reduction of 24.4 dB at frequencies of 8, 16, and 32 kHz. Control dabrafenib- and AZD5438-treated mice without noise exposure exhibited no significant threshold shift or change in body weight/behavior. Overall, this treatment regimen provided enhanced, nearly full protection compared to each compound alone at all frequencies tested (Fig. 6D).

## DISCUSSION

Noise-induced hearing loss is the second most common form of sensorineural hearing impairment after age-related hearing loss (presbycusis) in the United States (*56*). Cisplatin-induced hearing loss is an unavoidable side effect of chemotherapy with 40 to 60% of patients with cancer suffering from various degrees of hearing loss (*8*, *57*). Identifying drugs that can be given to patients during chemotherapy and after noise exposure to protect and prevent hearing loss is an urgent unmet medical need.

### Dabrafenib protects against cisplatin and noise damage

Dabrafenib, a BRAF-specific inhibitor, was a top hit in our cell line screen against cisplatin-induced cell death and showed ex vivo protection in cochlear explant cultures as well as significant in vivo protection in zebrafish lateral line neuromasts and in mouse models of cisplatin and noise-induced hearing loss. We used, in this study, a one-time high-dose cisplatin regimen (30 mg/kg) as proof of principle in mice to test dabrafenib’s protection from cisplatin. This one-dose cisplatin protocol in our hands correlates well with a low multi-dose cisplatin protocol (*58*) as we have shown previously for kenpaullone comparing both protocols (*13*). We tested the noise protection of dabrafenib at conditions that cause permanent hearing loss in FVB mice, 100-dB SPL octave band for 2 hours, and which are in the range of noise insults commonly experienced by people in everyday life. Together with the wealth of information of dabrafenib in human use, our results demonstrate that it can be rapidly repurposed for hearing protection and treatment in humans.

As we anticipated, dabrafenib crosses the blood-labyrinth barrier to have the observed effects in mice via oral delivery. Such properties of dabrafenib distinguish it from many other candidates currently in clinical trials for otoprotection (*1*, *2*, *59*) and further structural activity studies will provide insights into drug properties that are preferred for crossing the blood-labyrinth barrier (*60*).

### Dabrafenib does not interfere with cisplatin killing efficacy of tumor cells

In this study, we tested interference of dabrafenib with cisplatin killing efficacy in two representative tumor types in which cisplatin is given as part of the standard care—pediatric neuroblastoma and adult lung cancer. We found that in representative genetic cell lines of these tumors, dabrafenib does not interfere and in two cell lines, IMR-32 and SHP-77, even enhances the cisplatin tumor-killing efficacy at doses that protect from hearing loss in mice. Dabrafenib is well established as an effective drug against lung cancer (*18*), and drug treatment alone in this study suppressed, as expected, lung carcinoma cell survival with significant inhibition of SHP-77 cells, and a similar trend was observed in H115 and A549 cells (Fig. 4G). AZD5438 cotreatment with cisplatin did not interfere with cisplatin’s killing efficacy in five of the six cell lines tested, and even significantly enhanced the killing of three of the tumor cell lines (fig. S6C). In the neuroblastoma SKN-AS cell line, AZD5438 interfered with cisplatin killing ability, though when combined with dabrafenib, no interference was measured (fig. S6C). On the basis of dabrafenib’s chemical structure and its protective effects from noise ototoxicity, our evidence suggests that it does not directly interfere with cisplatin but acts through BRAF intracellular signaling. Such properties of dabrafenib make it superior to many other therapeutic candidates currently in clinical trials for protection against cisplatin ototoxicity.

### Dabrafenib protected against cisplatin- and noise-induced hearing loss in concentrations approved by the FDA for human use

The observed protective doses in mice are equivalent to those approved for human use. We did not observe whole-body toxicity or ototoxic effects to mice with 3-day dabrafenib-alone treatment by weight, general appearance/behavior, and ototoxicity up to 21 days (Figs. 4 and 5). When administered chronically, daily for 6 to 12 months to humans, in equivalent doses used here in mice, dabrafenib has side effects such as fever, joint pain, skin rash, and papilloma. It is therefore important to further determine in humans the side effects of dabrafenib in short-term usage, as well as the lowest effective dose in mice to determine the in vivo TI. Moreover, modifying dosing regimen and formulation would further enhance the clinical potential of dabrafenib as an otoprotectant in clinics. Combination therapy of dabrafenib/AZD5438, as we demonstrated here against noise-induced hearing loss, has the clear advantage of allowing for use of lower dosages of each drug that can reduce undesirable side effects without compromising protection or treatment efficacy.

### Dabrafenib inhibits phosphorylation of ERK kinase in the SCs

Dabrafenib inhibits a new cochlear target BRAF kinase and its associated downstream kinases MEK1/2 and ERK1/2, for protection from cell death induced by cisplatin and noise, suggesting that they have key proapoptotic roles in postmitotic cochlear cells. We observed up-regulation of phospho-ERK within a short time of both cisplatin and noise insults in the SCs, in particular the DCs and IPhCs, of the cochlea. The up-regulation of this BRAF/MEK/ERK pathway by both insults in overlapping cells not only explained why the same drug can protect from damage from both sources but also further validated our screen methodology for identifying drugs that shield from both insults. Germline knockout of BRAF in mice causes death of embryos between E10.5 and 12.5 owing to growth retardation and vascular and neuronal defects Wojnowski *et al*. (*61*). Generating a mouse model in which BRAF is specifically deleted in the inner ear cells and/or the cochlear SCs will further assess the mechanisms by which BRAF plays a proapoptotic role in postmitotic cochlear cells.

The important role of the SCs in triggering and relaying cell death signals in the cochlea has been shown by several studies in recent years. Lahne and Gale showed that within minutes of subjecting cochlear explants to mechanical damage or the aminoglycoside neomycin, cytoplasmic phospho-ERK was transiently activated in Deiters’ and phalangeal cells but not HCs (*26*). Maeda *et al*. (*27*) and Herranen *et al*. (*28*) showed that nuclear phospho-ERK signaling was transiently activated in the supporting cochlear cells after 2-hour 120- or 105-dB octave-band noise. Our data, combined with previous publications, suggest that there is activation of pERK in the cytoplasm immediately after various insults such as cisplatin, noise, aminoglycoside, or mechanical damage, and pERK translocates to the nucleus a few hours later. ERK protein is known to shuttle to the nucleus to exert its activity (*62*). In addition, in adult cochleae, we observed pERK staining in the areas of stria vascularis and nerve fiber bundle innervating the HCs 1 hour after cisplatin administration that was down-regulated by dabrafenib (Fig. 4F). Previous reports have documented cisplatin-induced damage to the stria and spiral ganglion neurons, reinforcing these cells as sensitive to cisplatin treatment (*63*–*66*).

Our results suggest that downstream targets of pERK activation in cells can regulate nuclear transcription factors that increase transcription of secreted ligands to surrounding cells as the HCs and thus affect their survival. The protective role of secreted heat shock protein HSP70 from the vestibular SCs in the death pathway after antibiotic insults has been reported (*67*, *68*). The ERK kinase up-regulates heat shock proteins that are involved in the stress response pathway in several postmitotic cell systems (*69*). Also, ERK phosphorylation can trigger immune response in the cochlea and may contribute to recruitment of activated macrophages to the damaged area (*28*, *70*). Thus, our studies here suggest that BRAF/MEK/ERK and CDK2 pathways are upstream regulators of HSP70 and macrophage recruitment to mediate their stress-activating effects in postmitotic cells.

### Protective mechanisms of BRAF inhibition in postmitotic cells

When activated in tumor cells, the BRAF/MEK/ERK pathway is commonly associated with cell proliferation and survival. In contrast, in cochlear postmitotic cells, inhibition of the BRAF pathway by dabrafenib reduces cell death and enhances cell survival as shown here. CDK2 inhibitors that we previously identified in similar screens also showed protection in postmitotic cochlear cells whereas both BRAF inhibitors and CDK2 inhibitors are two known groups of anti-proliferative compounds in tumor cells (*13*, *34*). Cellular roles for ARAF (Raf-1), a family member of BRAF, in protection from apoptotic stimuli have been shown previously in endothelial and cardiomyocyte postmitotic cells, although these pathways were independent of MEK kinase (*71*, *72*). We therefore propose that BRAF/MEK/ERK and CDK2 play proapoptotic roles in postmitotic cells while playing pro-proliferative roles in tumor cells. It remains to be explored how these pathways are activated upon stress upstream and how their pharmacological inhibitions mitigate apoptosis downstream in postmitotic cells. Epistasis between BRAF/MEK/ERK and CDK2 and their pathways can be further analyzed using pharmacogenetic interventions in vivo with or without cisplatin or noise exposure.

### Therapeutic potentials of orally delivered dabrafenib and AZD5438

In summary, our study provides key results required for repurposing dabrafenib as a candidate therapeutic drug for protection against cisplatin- and noise-induced hearing loss. The combination of dabrafenib with AZD5438 conferred full protection even when given 24 hours after noise exposure. On the basis of our results, dabrafenib can also be tested for antibiotic-induced and age-related hearing loss in the inner ear (*2*, *6*, *59*, *73*, *74*). Our studies will hopefully lead to one of the first FDA-approved drugs repurposed specifically for hearing protection (*75*). We also provide evidence that BRAF/MEK/ERK and CDK2 pathways, while key in tumor cells, play proapoptotic and stress-activating roles in postmitotic cochlear cells, an underappreciated feature of these pathways in postmitotic cells. As a result, dabrafenib could also be useful for treating degenerative diseases afflicting postmitotic cells in the kidney and brain. Dabrafenib came up recently as a top hit in a silico drug screen for treating Parkinson’s disease (*76*).

## MATERIALS AND METHODS

### Ethics statement

All animal procedures were approved by the Institutional Animal Care and Use Committee of Creighton University and Boys Town National Research Hospital.

### Mouse models

FVB breeding mice were purchased from the Jackson Laboratory, bred in the Creighton University animal facility, and used for the noise damage and cisplatin treatment experiments. Animals were anesthetized by Avertin (500 mg/kg) intraperitoneal injection (2,2,2-Tribromoethanol, T4, 840-2; Sigma-Aldrich), and complete anesthetization was determined via toe pinch. Anesthetized animals were kept on heating pads to maintain body temperature throughout experimental procedures until they awaken.

### Zebrafish models

Zebrafish (*Danio rerio*) line Tg(brn3c:GFP) was obtained by pair mating of adult fish maintained at Boys Town National Research Hospital by standard methods approved by the Institutional Animal Care and Use Committee.

### Cell lines

Three human neuroblastoma cell lines IMR-32, SH-SY5Y, and, SK-N-AS, as well as three human lung carcinoma cell lines SHP-77 small cell, H1155 non–small cell, and A549 non–small cell, were used to determine the compounds’ effect on cisplatin’s tumor-killing efficacy (*77*–*83*). Cell lines were purchased from the American Type Culture Collection (ATCC), and cell culture was conducted according to ATCC specifications. Neuroblastoma cell lines IMR-32 and SH-SY5Y are maintained in Dulbecco’s modified Eagle’s medium (DMEM) 1× with glucose (4.5 g/liter), l-glutamine, and sodium pyruvate (10-013-CV, Corning) with 10% fetal bovine serum (FBS) (35011132, Corning) and ampicillin (50 μg/ml; A5354-10ML, Sigma-Aldrich) added. The SK-N-AS cell line is maintained in DMEM 1× with d-glucose (4.5 g/liter), l-glutamine, and 25 mM Hepes (12430-054, Gibco) with 10% FBS, 0.1 mM nonessential amino acids, and ampicillin (50 μg/ml) added. A549 cells are maintained in F-12K medium with l-glutamine (30-2004, ATCC) with 10% FBS and ampicillin (50 μg/ml) added. SHP-77 and H1155 cell lines are maintained in RPMI 1640 (30-2020, ATCC) with 5% FBS and ampicillin (50 μg/ml) added. All tumor cell lines are grown at 37°C and 5% CO_2_ and passaged using 0.05% trypsin-EDTA 1× (25300-054, Gibco). The HEI-OC1 cell line (*12*–*14*) is maintained in DMEM 1× with glucose (4.5 g/liter), l-glutamine, and sodium pyruvate (12430-054, Gibco) with 10% FBS, ampicillin (50 μg/ml), and 250 μl of γ-interferon added. These cells are cultured under permissive conditions 33°C and 10% CO_2_ and passaged using 0.05% trypsin-EDTA 1×. For HEI-OC1 cells, 24 hours before experimental procedures, γ-interferon is removed from medium to limit cell growth.

### Cell-based caspase-3/7 activity and Cell Titer-Glo viability HTSs for protection from cisplatin-induced cell death

In these screens, caspase-3 cleavage was chosen as the end point indicating cisplatin-induced apoptosis, because it allowed the inhibition of cell death to be monitored at the level of any molecular target upstream of caspase-3 cleavage in the HEI-OC1 cell line. Cells were plated at a previously optimized concentration of 1600 cells per well in medium without γ-interferon to limit proliferation and tested against 50 μM cisplatin based on a previous dose–response curve. Tested compounds were dissolved in DMSO before addition to medium with a final DMSO concentration of <0.5%. The screens are described in detail in our previous publications (*12*, *13*). Pifithrin-α was chosen as a reference compound for the screen, as it provided good protection against cisplatin ototoxicity by inhibiting caspase-3 cleavage with an IC_50_ of 7.7 μM (fig. S1) (*5*, *12*, *84*). Pifithrin-α was added to each plate as a screening quality control. The linearity of the Caspase-Glo 3/7 (Promega) assay, which enables the measurement of light emitted as a result of caspase-3/7 cleavage and is suitable for HTS, was validated, and it was verified that 0.5% DMSO had no effect on the cell death kinetics. Compounds were screened in triplicate at final concentrations ranging from 1 nM to 85 μM and incubated for 22 hours. The Caspase-Glo 3/7 assay defined 100% caspase-3/7 activity as cells treated with cisplatin alone and cells treated with medium alone assigned 0% caspase-3/7 activity. Hits were defined as compounds that reduce caspase-3/7 activity by 50% or more in the presence of 50 μM cisplatin. Compound toxicity was tested by a separate viability assay Cell Titer-Glo (Promega) with compound alone (*12*). The dataset of protein kinase inhibitors screened has been deposited at https://data.mendeley.com/datasets/yz9rbxyksf/1, DOI: 10.17632/yz9rbxyksf.1.

### Tumor Cell Titer-Glo viability assay

Cells were initially plated in 96-well plates and allowed to attach overnight at 37°C in 5% CO_2_. The following day, cancer cells were pretreated with compound alone for 1 hour, followed by 15 μM cisplatin (200 μM for A549) plus compound treatment for 48 hours and then assayed via Cell Titer-Glo (Promega) to measure viability. Medium-alone–, cisplatin-alone–, and drug-alone–treated cells were used as controls, and viability was reported as percent survival compared to medium alone. Dabrafenib (35 μM) and AZD5438 (2.1 μM), three times the protective IC_50_ against cisplatin in the caspase-3/7 HTS, were used.

### Cochlear explants

P3-P4 FVB mouse cochleae were dissected and maintained in culture on filter inserts in six-well plates with 1 ml of growth medium DMEM (12430-054,Gibco Life Technologies), with 1% FBS (16000-044, Gibco Life Technologies), B-27 supplement (200 μl/500 ml; 17504-44, Gibco Life Technologies), N-2 supplement (100 μl/500 ml; 17502-048, Gibco Life Technologies), and ampicillin (50 μg/ml; A5354-10ML, Sigma-Aldrich) both inside and outside the filter (Millicell, PICM03050, Millipore). After cochlear explants had been in culture for 1 day, growth medium with or without the test compound was added for preincubation for 1 hour at 37°C in 5% CO_2_, followed by incubation with 150 μM cisplatin (479306, Sigma-Aldrich) with or without the test compound in growth medium for 24 hours at 37°C. A cisplatin concentration of 150 μM was chosen on the basis of the dose responses of cisplatin at 50, 100, 150, and 200 μM and because the explant assay consistently showed that ∼40% of OHCs in the mouse cochlea died at this concentration after 24 hours (Figs. 1D and 2, B to H). Cochlea were fixed with 4% paraformaldehyde and stained for F-actin with Alexa Fluor 568 phalloidin to determine the viability of the HCs. Cochleae were imaged by confocal microscopy, two 160-μm regions from middle turns were photographed, and the number of intact HCs was counted. Two to five independent cochleae were tested for each experimental condition. Note that the IC_50_ of dabrafenib in the ex vivo explant culture is 400-fold better than the IC_50_ measured in the inner cell line and may be attributed to specific receptors for the drug that are present only in the cochlear explant culture cells. This was not unique to dabrafenib and was observed with all our top hits in this study and our previous studies (Fig. 2 and table S1) (*13*).

### Zebrafish lateral lines

Zebrafish (*D. rerio*) experimental larvae of either sex were obtained by pair mating of adult fish maintained at Boys Town National Research Hospital. We used zebrafish line Tg(brn3c:GFP) that expresses a membrane-bound GFP in HCs. Fish were maintained at 28.5°C in E3 media [5 mM NaCl, 0.17 mM KCl, 0.33 mM CaCl_2_, and 0.33 MgSO_4_ (pH 7.2)] at a density of 50 embryos/larvae per 100-mm^2^ petri dish. Experimental animals were used at 5 days post-fertilization and cryo-anesthetized after treatment and before fixation. Average HC counts in our studies were obtained from the following three neuromasts: MI1 (medial neuromast 1) and O1 and O2 (otic line).

### Cisplatin treatment in mice

Ten milligrams of cisplatin (479306, Sigma-Aldrich) powder was dissolved in 10 ml of sterile saline (0.9% NaCl) at 37°C for 40 to 60 min. Cisplatin was administered to FVB mice at 30 mg/kg via intraperitoneal injection. One day before cisplatin injection, mice received 1 ml of saline by subcutaneous injection. To ameliorate dehydration after cisplatin injection, 1 ml of warm saline was injected twice per day for at least 7 days or until body weight started to recover. The cages of cisplatin-treated mice were placed on the heating pad for at least 7 days or until body weight began to recover. Fresh mush food was daily given for at least 7 days. A survival rate of 50% was observed in both cisplatin and combined cisplatin with dabrafenib treatments.

### Compound administration by oral gavage

Compounds dabrafenib mesylate (HY-14660A) and AZD5438 (HY-10012) were purchased from MedChemExpress and administered to FVB mice via oral gavage. Compounds were dissolved in a mixture of 10% DMSO, 5% Tween 80 (P1754, Sigma-Aldrich), 40% PEG-E-300 (91462-1KG, Sigma-Aldrich), and 45% 0.9% saline. For cisplatin and noise pretreatment protocols, mice were given initial treatments of dabrafenib (100 mg/kg) and AZD5438 (75 mg/kg) 45 min before ototoxic insult, and follow-up treatments were given 24 and 48 hours after insult. For noise posttreatment protocol, compound treatments were administered twice daily at dabrafenib (60 mg/kg) and AZD5438 (35 mg/kg) 8 hours apart at 24-, 48-, and 72-hour time intervals after noise insult.

### ABR threshold and wave 1 amplitude measurements

ABR in mice was measured for left ears as described previously with minor modifications (*13*, *34*). In brief, ABR waveforms were recorded in a sound booth (Industrial Acoustic Company) by using subdermal needles positioned in the skull, below the pinna, and at the base of the tail, and the responses were fed into a low-impedance Medusa digital biological amplifier system (RA4L; TDT; 20-dB gain). At each frequency, the stimulus intensity was reduced from 90 to 0 dB in 10-dB steps to determine the threshold decibel SPL when the electrical response was just above the noise floor. ABR waveforms were averaged in response to 500 tone bursts. The recorded signals were filtered by a band-pass filter from 300 Hz to 3 kHz. Pretreatment ABR recordings of adult FVB mice (aged 6 weeks) were acquired 1 week before all treatments, and posttreatment ABR measurements were recorded 21 days after cisplatin treatment or 14 days after noise exposure. ABR threshold was determined by the presence of any of three or more of the five waveform peaks. Base ABR threshold values for mice used in experiments were between 10 and 40 dB at all tested frequencies. All thresholds and shifts were determined independently by two experimenters for each mouse. Individual ABR wave 1 amplitudes were measured as the difference between the positive peak and the following negative trough.

### Noise exposure in mice

Mice were placed in individual cages in a custom-made acrylic chamber. The sound stimulus was produced by System RZ6 (TDT) equipment and amplified using a 75-A power amplifier (Crown). Sound was delivered to the acrylic chamber via a speaker horn (JBL). The SPL was calibrated with a 1/4-inch free-field microphone (PCB). Before experimental noise exposure, four quadrants of the cage inside the chamber were sampled with the 1/4-inch microphone to ensure that the SPL varied by <0.5 dB across the measured positions. Adult mice were then exposed to 2 hours of octave band noise (8 to 16 kHz) at 100 dB.

### Tissue preparation and immunofluorescence

Cochleae from adult mice were prepared and examined as described previously (*85*, *86*). All images were acquired with a confocal microscope (LSM 700 or 710, Zeiss). Samples were stained with phalloidin (A12379 or A12380, Invitrogen) or with antibodies for other markers. The following primary antibodies were used: anti–myosin VI (1:400; 25-6791; Proteus Bioscience), anti–myosin VIIa (5 μg/μl;138-1-s, Developmental Studies Hybridoma Bank), anti-Tuj1 (1:250; 801201, BioLegend), anti-pERK (1:400; 9101S, Cell Signaling Technology), or anti-Ctbp2 (1:500; 612044, BD Transduction). Anti-rabbit Alexa Fluor 488 (1:400; A11034) and anti-mouse Alexa Fluor 647 (1:400; A31571) secondary antibodies were purchased from Invitrogen. ProLong gold antifade reagent with 4′,6-diamidino-2-phenylindole (P36941, Invitrogen) was used to counterstain nuclei.

### Ctbp2 staining and quantification

Organs of Corti were costained with anti-Ctbp2 and anti–myosin VI antibodies. Confocal imaging was performed using a Zeiss 700 scanning confocal microscope with a 63× objective. Visualization and projections were performed using ZEN 2009 or ZEN 2012 software (Zeiss). Ctbp2 puncta were visualized and counted on the reconstructed three-dimensional images by using the ZEN 2009 or ZEN 2012 three-dimensional construct function as previously reported (*54*). Cochleae from two dabrafenib-alone–treated FVB mice without noise exposure and cochleae from four carrier and four dabrafenib-treated FVB mice exposed to noise were randomly chosen from experimental cohorts and used to quantify the Ctbp2 puncta. Images were acquired around 16-kHz cochlear regions, and each image included 12 to 18 IHCs that were quantified as total number of Ctbp2 puncta divided by total number of IHCs per image.

### Immunoblotting

Whole lysates of HEI-OC1 cells were prepared in lysis buffer (9803, Cell Signaling Technology) after adding protease (cOmplete ULTRA Tablets 05892791001) and phosphatase (PhosSTOP 04906845001) inhibitors (Roche). The lysates were centrifuged for 10 min at 15,000*g* at 4°C, and the supernatants were collected. Protein concentrations in protein solutions were determined with the BCA protein assay kit (23235, Thermo Fisher Scientific). Forty micrograms of total cell lysate was loaded on 4 to 20% SDS–polyacrylamide gel electrophoresis gels. The following antibodies were used for immunoblot analysis: anti-BRAF (14814S), anti-pBRAF (Ser^445^, 2696S), anti-ERK1/2 (4695), anti-pERK1/2 (Thr^202^/Tyr^204^, 9101S), anti-MEK1/2 (9122S), and anti-pMEK1/2 (Ser^217^/221, 41G9S) were obtained from Cell Signaling Technologies and anti–β-actin (C4; SC-47778) was purchased from Santa Cruz. The antibodies were used at dilutions ranging from 1:500 to 1:1000. Anti-mouse (P0447) and anti-rabbit (P0448) secondary antibodies were purchased from Dako Laboratories and diluted 1:5000. National Institutes of Health (NIH) ImageJ software was used to quantify the band intensities and recorded as a ratio to loading control β-actin. Each Western blot series (n) was carried out using cell lysates from separate experiments. Blot intensities were quantified using NIH ImageJ software.

### Statistical analysis

Statistics was performed by using Prism (GraphPad Software). We used one-way or two-way analyses of variance (ANOVAs) with Bonferroni post hoc test for mean difference or a two-sample Student’s *t* test if only two conditions were compared.

## Supplementary Material

http://advances.sciencemag.org/cgi/content/full/6/49/eabd0561/DC1

Adobe PDF - abd0561_SM.pdf

BRAF inhibition protects against hearing los in mice

## SUPPLEMENTARY MATERIALS

Supplementary material for this article is available at http://advances.sciencemag.org/cgi/content/full/6/49/eabd0561/DC1

## Appendix

View/request a protocol for this paper from *Bio-protocol*.
