# Supplementary material for: BRAF inhibition protects against hearing loss in mice
Source: Sci Adv. 2020 Dec 2;6(49):eabd0561. doi: 10.1126/sciadv.abd0561 (PMC7821884; doi:10.1126/sciadv.abd0561)
Supplement: http://advances.sciencemag.org/cgi/content/full/6/49/eabd0561/DC1 [file supp_6_49_eabd0561__index.html]

Science Advances | Science AdvancesAAASSearchScience AdvancesMenu

## Supplementary Materials

# BRAF inhibition protects against hearing loss in mice

Matthew A. Ingersoll, Emma A. Malloy, Lauryn E. Caster, Eva M. Holland, Zhenhang Xu, Marisa Zallocchi, Duane Currier, Huizhan Liu, David Z.Z. He, Jaeki Min, Taosheng Chen, Jian Zuo, Tal Teitz

Download Supplement

**This PDF file includes:**

- Figs. S1 to S8
- Table S1

**Files in this Data Supplement:**

- Adobe PDF - abd0561\_SM.pdf
