# Supplementary material for: BRAF inhibition protects against hearing loss in mice
Source: Sci Adv. 2020 Dec 2;6(49):eabd0561. doi: 10.1126/sciadv.abd0561 (PMC7821884; doi:10.1126/sciadv.abd0561)
Supplement: http://advances.sciencemag.org/cgi/content/full/6/49/eabd0561/DC1 [file supp_6_49_eabd0561__1.pdf]

Science Advances
AAAS

advances.sciencemag.org/cgi/content/full/6/49/eabd0561/DC1

Supplementary Materials for

BRAF inhibition protects against hearing loss in mice

Matthew A. Ingersoll, Emma A. Malloy, Lauryn E. Caster, Eva M. Holland, Zhenhang Xu, Marisa Zallocchi, Duane Currier, Huizhan Liu, David Z.Z. He, Jaeki Min, Taosheng Chen, Jian Zuo, Tal Teitz*

*Corresponding author. Email: talteitz@creighton.edu

Published 2 December 2020, *Sci. Adv.* **6**, eabd0561 (2020)
DOI: 10.1126/sciadv.abd0561

This PDF file includes:

Figs. S1 to S8
Table S1

SUPPLEMENTARY MATERIALS

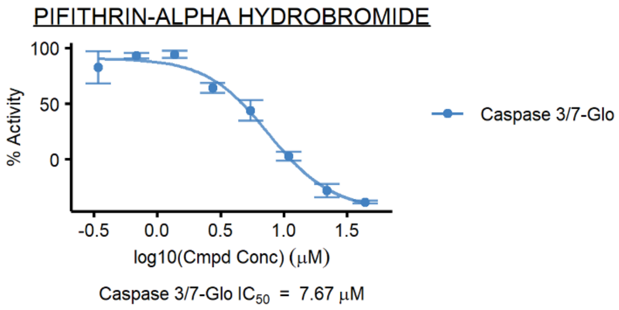

Supplemental Figure 1 – Dose-response Caspase-Glo- 3/7 assay for the reference compound in the HEI-OC1 cell line screen, related to Figure 1.

A representative 3/7 Caspase-Glo dose-response curve for the reference compound pifithrin-alpha hydrobromide that was present in each plate in the cell-based compound screen.

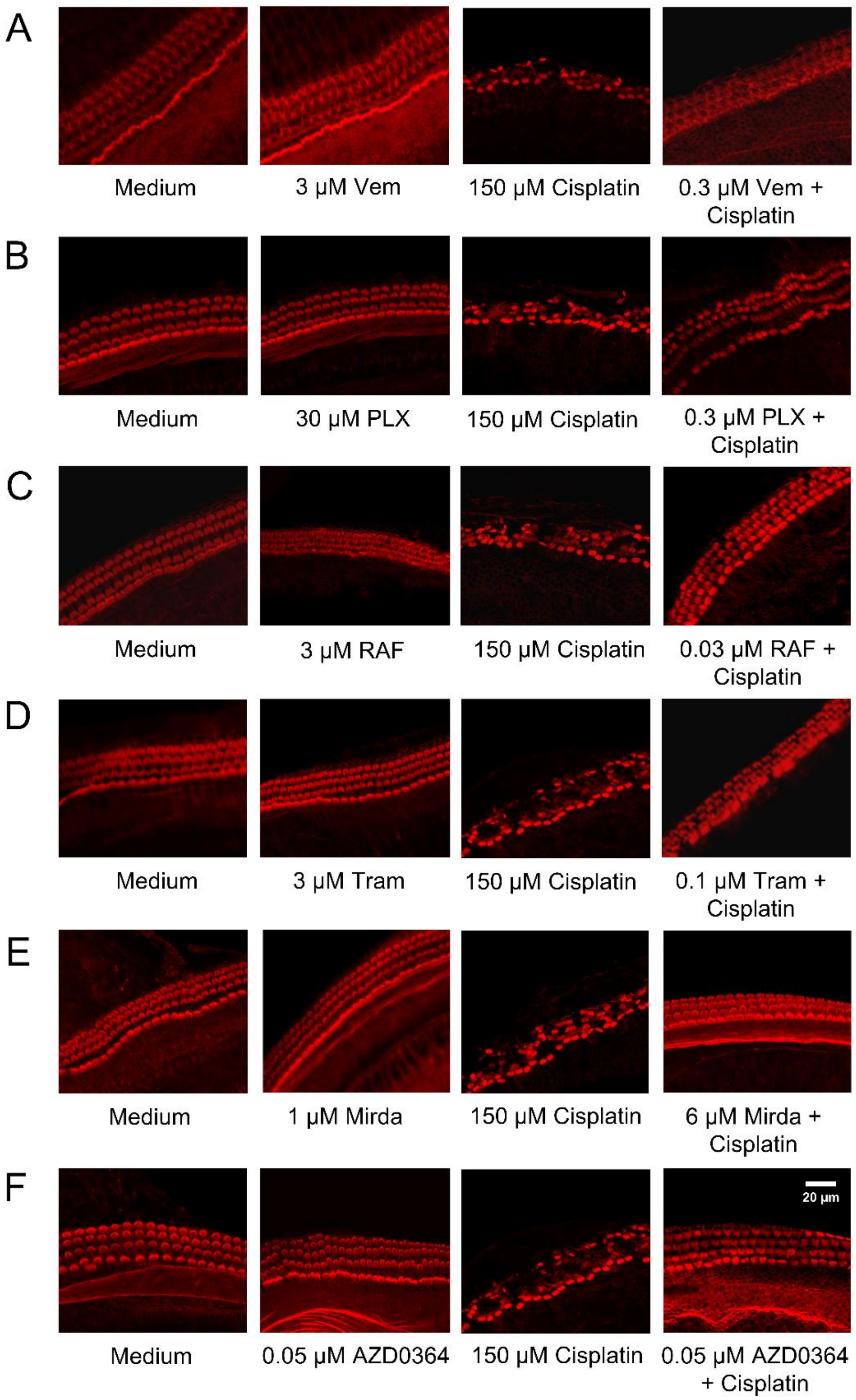
A
Medium
3 μM Vem
150 μM Cisplatin
0.3 μM Vem + Cisplatin
B
Medium
30 μM PLX
150 μM Cisplatin
0.3 μM PLX + Cisplatin
C
Medium
3 μM RAF
150 μM Cisplatin
0.03 μM RAF + Cisplatin
D
Medium
3 μM Tram
150 μM Cisplatin
0.1 μM Tram + Cisplatin
E
Medium
1 μM Mirda
150 μM Cisplatin
6 μM Mirda + Cisplatin
F
Medium
0.05 μM AZD0364
150 μM Cisplatin
0.05 μM AZD0364 + Cisplatin
20 μm

Supplemental Figure 2 – Confocal images of BRAF, MEK1/2, and ERK1/2 inhibitors tested in mouse cochlear explants, related to Figure 2.

Phalloidin stained confocal images of P3 FVB mouse cochlear explants pre-treated with drug alone for 1 h followed by 24 h drug treatment with or without 150 μM cisplatin. From left to right: Medium alone, drug alone (maximum tested non-toxic concentration), cisplatin alone, drug + cisplatin treatment (maximum protective concentration). (A) Vemurafenib, BRAF inhibitor. (B) PLX-4720, BRAF inhibitor. (C) RAF-265, BRAF inhibitor. (D) Trametinib, MEK1/2 inhibitor. (E) Mirdametinib, MEK1/2 inhibitor. (F) AZD0364, ERK1/2 inhibitor.

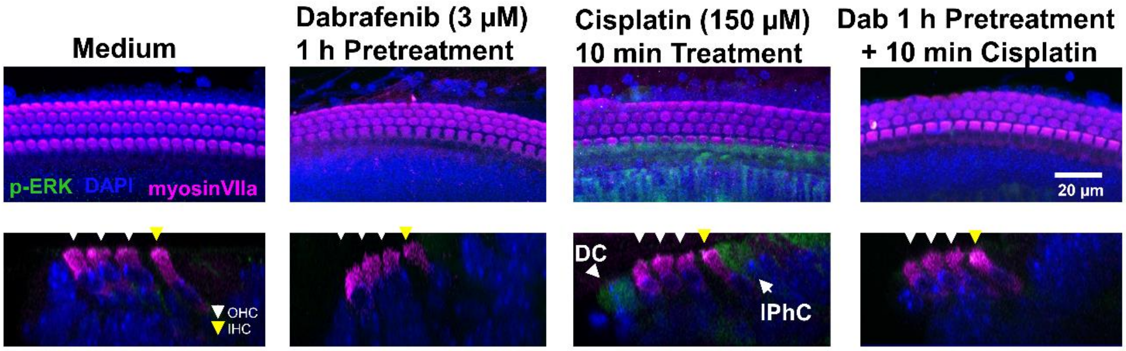

Supplemental Figure 3 – Confocal images of cochlear explants stained for phosphorylated ERK post cisplatin treatment, related to Figure 3.

Confocal images of cochlear explants treated with dabrafenib and cisplatin. DAPI (blue), phosphorylated ERK (green), and myosin7a (pink) stained confocal images of P3 FVB whole-mount middle turn mouse cochlea explants pre-treated with 3 µM dabrafenib (Dab) for 1 h before 10 min cisplatin (150 µM) exposure. Ortho section shown below in which outer hair cells (OHC) are identified with white arrows, inner hair cells (IHC) with yellow arrows, and phosphorylated ERK positive Dieters' cells (DC) and inner phalangeal cells (IPhC) with labeled arrows. n = 6 cochlea.

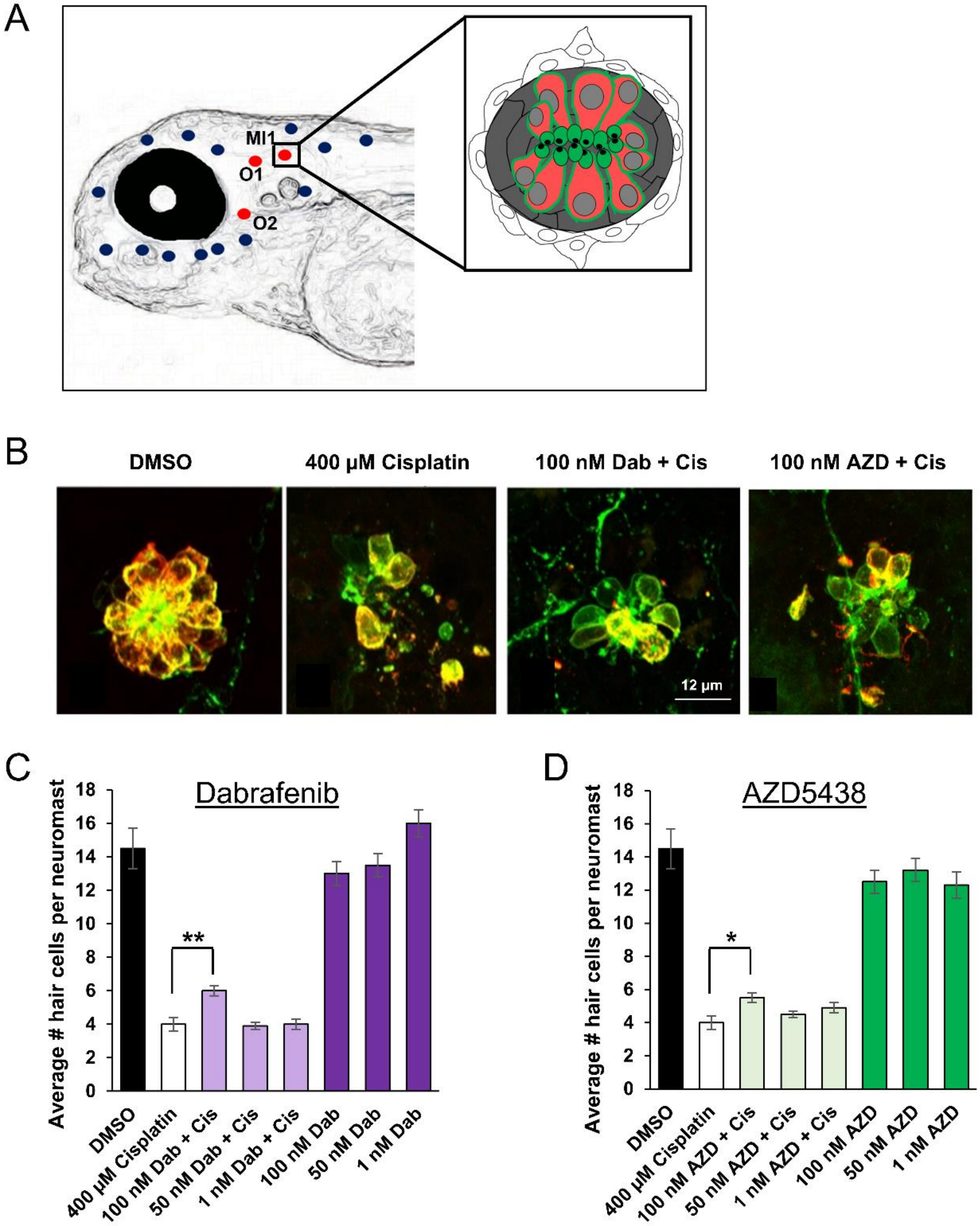
A
MI1
O1
O2
B
DMSO
400 μM Cisplatin
100 nM Dab + Cis
100 nM AZD + Cis
12 μm
C
Dabrafenib
Average # hair cells per neuromast
0
2
4
6
8
10
12
14
16
18
**
DMSO
400 μM Cisplatin
100 nM Dab + Cis
50 nM Dab + Cis
1 nM Dab + Cis
100 nM Dab
50 nM Dab
1 nM Dab
D
AZD5438
Average # hair cells per neuromast
0
2
4
6
8
10
12
14
16
18
*
DMSO
400 μM Cisplatin
100 nM AZD + Cis
50 nM AZD + Cis
1 nM AZD + Cis
100 nM AZD
50 nM AZD
1 nM AZD

Supplemental Figure 4 – Dabrafenib and AZD5438 confer protection against cisplatin induced HC loss in zebrafish in vivo.

(A) Overview of the zebrafish lateral line system and the neuromast cells. Lateral line view of a 5dpf larva showing the approximate location of the neuromasts inspected in our studies in red and additional neuromasts in blue. Schematic drawing of a top view of a neuromast showing the hair cells (red), supporting cells (grey) and mantle cells (white). MI1: medial neuromast 1, O1: Otic neuromast 1 and O2: Otic neuromast 2. (B) Five days post-fertilization Tg(brn3c: GFP) zebrafish were treated with dabrafenib (Dab) or AZD5438 (AZD) alone or pre-treated with compound for 1 h, followed by 6 h 400 μM cisplatin and allowed to recover for 1 h in fresh water. Fish were fixed and stained for GFP (green) and otoferlin (red) to identify neuromast HCs. The average number of HCs at MI1 and O1-2 were counted from at least three animals. Confocal representative images of MI1 are shown in (A), dabrafenib treatment counts in (B), and AZD5438 treatment counts in (C). Medium alone (black bar) and cisplatin alone (white bar) are used as controls. Mean ± SEM, P=*<0.05, **<0.005 compared to cisplatin alone by unpaired two-tailed Student's t-test.

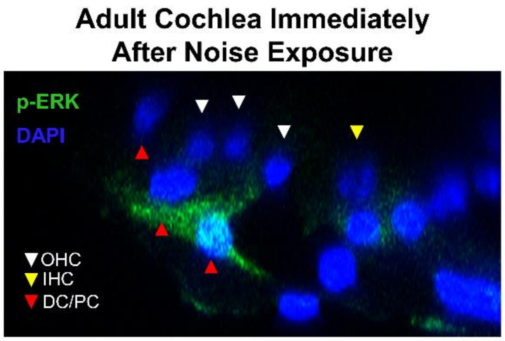

Supplemental Figure 5 – Confocal images of adult cochlea stained for phosphorylated ERK post noise exposure, related to Figure 5.

Confocal ortho section of adult mouse cochlea collected and fixed immediately after 8-16 kHz octave-band noise exposure for 2 h and stained with DAPI (blue) and phosphorylated ERK (green). OHC identified with white arrows, IHC with yellow, and DC and PC with red. n = 2 cochlea.

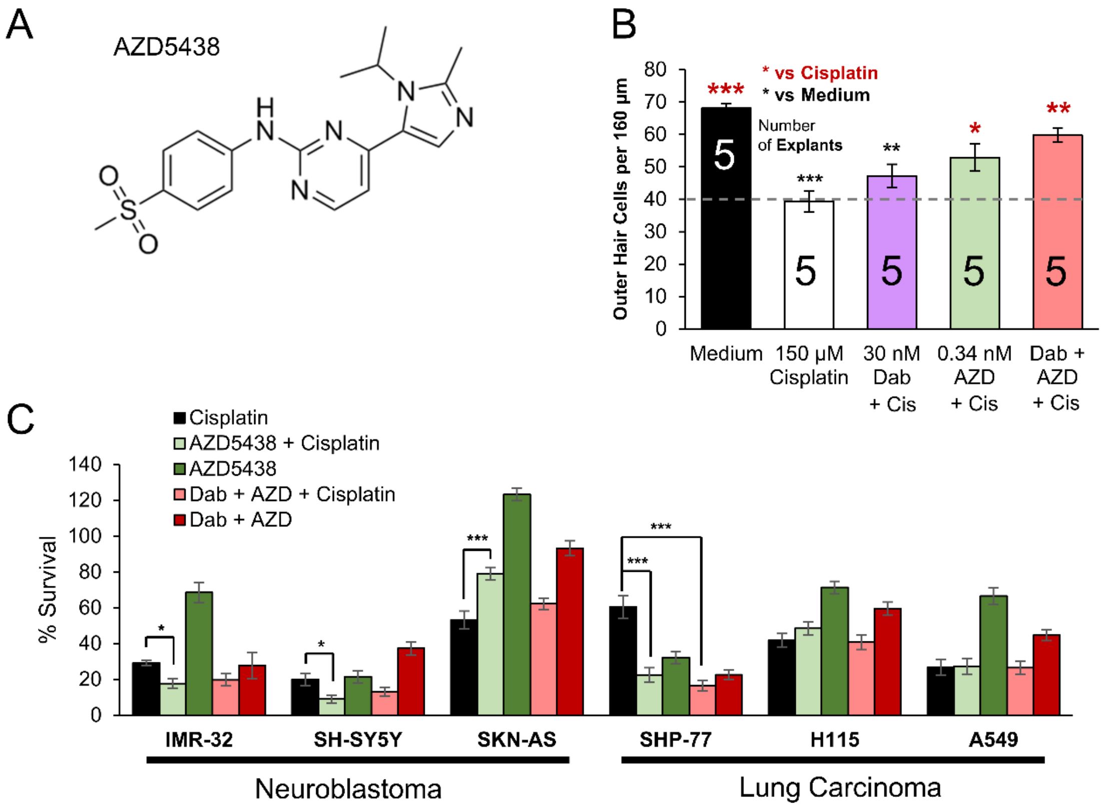

A
AZD5438
B
* vs Cisplatin
* vs Medium
Number of Explants
Outer Hair Cells per 160 µm
Medium
150 µM Cisplatin
30 nM Dab + Cis
0.34 nM AZD + Cis
Dab + AZD + Cis
C
Cisplatin
AZD5438 + Cisplatin
AZD5438
Dab + AZD + Cisplatin
Dab + AZD
% Survival
IMR-32
SH-SY5Y
SKN-AS
SHP-77
H115
A549
Neuroblastoma
Lung Carcinoma

<u>Supplemental Figure 6</u> – AZD5438 and dabrafenib combined protective effect in mouse cochlear explants, related to Figure 6.

(A) Molecular structure of AZD5438. (B) P3 FVB mouse cochlear explants were pre-treated with drug alone for 1 h followed by 24 h drug + 150 µM cisplatin treatment. Medium alone (black), cisplatin alone (white), 30 nM dabrafenib + cisplatin (purple), 0.34 nM AZD5438 + cisplatin (green), combined 30 nM dabrafenib and 0.34 nM AZD5438 + cisplatin (red). Number of outer HCs per 160 µm of middle turn regions of the cochlea were counted by phalloidin staining, mean ± SEM, P=*<0.05, **<0.005, ***<0.0005 compared to cisplatin alone (red) and medium alone (black) by one-way ANOVA with Bonferroni post hoc test. (C) Cell Titer-Glo percent cell survival of neuroblastoma and lung carcinoma cell lines pretreated 1 h with drug followed by 48 h combined cisplatin and drug treatment. Cisplatin (black), AZD5438 + cisplatin (light green), AZD5438 alone (dark green), combined dabrafenib and AZD5438 + cisplatin (light red), combined dabrafenib and AZD5438 alone (dark red), Data shown as mean ± SEM, P=*<0.05, **<0.01, ***<0.001 compared to cisplatin alone (red) and medium alone (black) by one-way ANOVA with Bonferroni post hoc test.

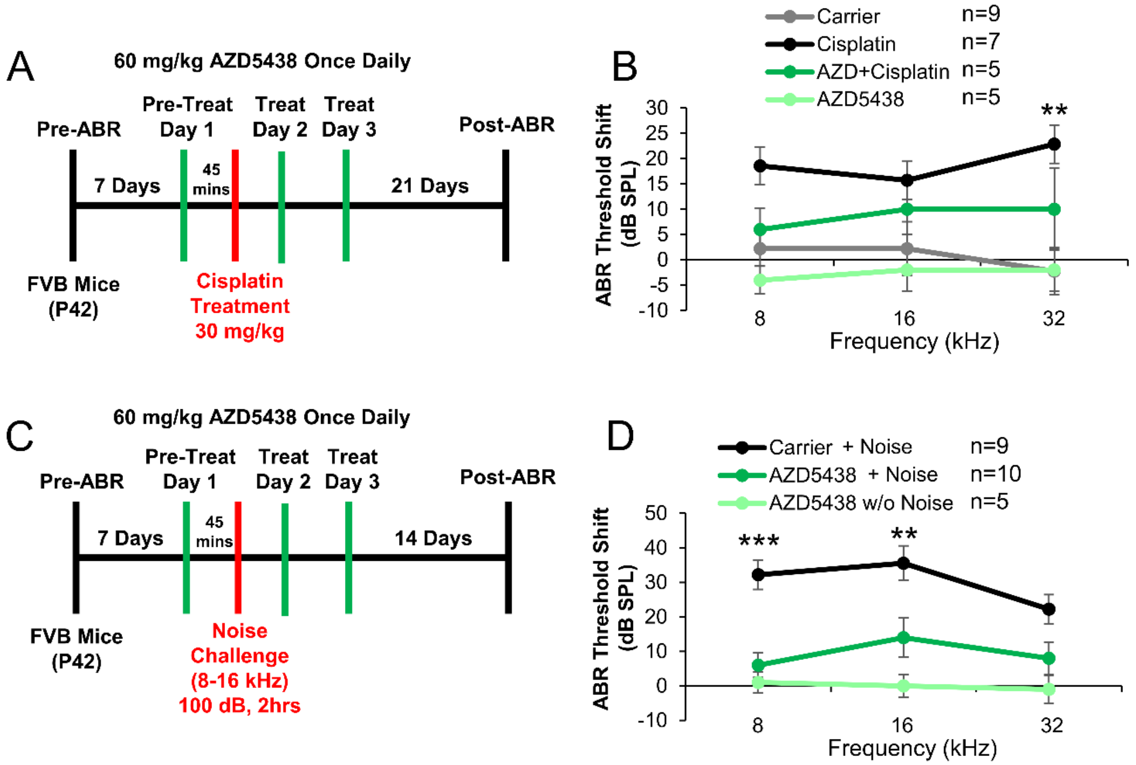
A
60 mg/kg AZD5438 Once Daily
Pre-ABR
Pre-Treat Day 1
Treat Day 2
Treat Day 3
Post-ABR
7 Days
45 mins
21 Days
FVB Mice (P42)
Cisplatin Treatment 30 mg/kg
B
Carrier n=9
Cisplatin n=7
AZD+Cisplatin n=5
AZD5438 n=5
**
ABR Threshold Shift (dB SPL)
Frequency (kHz)
C
60 mg/kg AZD5438 Once Daily
Pre-ABR
Pre-Treat Day 1
Treat Day 2
Treat Day 3
Post-ABR
7 Days
45 mins
14 Days
FVB Mice (P42)
Noise Challenge (8-16 kHz) 100 dB, 2hrs
D
Carrier + Noise n=9
AZD5438 + Noise n=10
AZD5438 w/o Noise n=5
***
**
ABR Threshold Shift (dB SPL)
Frequency (kHz)

Supplemental Figure 7 – AZD5438 confers protection from cisplatin and noise induced hearing loss, related to Figure 6.

(A) Schedule of administration of AZD5438 and cisplatin to adult P42 FVB mice. (B) ABR threshold shifts recorded 21 days after 30 mg/kg cisplatin by IP injection and 75 mg/kg AZD5438 treatment by oral gavage. Untreated controls (grey), cisplatin alone (black), AZD5438 alone (light green), AZD5438 and cisplatin (dark green). Mean ± SEM, P=*<0.05, **<0.01, compared to cisplatin alone by two-way ANOVA with Bonferroni post-test. (C) Schedule of administration of dabrafenib and noise-exposure to adult P42 FVB mice. (D) ABR threshold shifts recorded 14 days after 100 dB 8-16 kHz octave band noise for 2 h for control and 75 kg/mg AZD5438 once daily treated mice by oral gavage. AZD5438 treatment without noise exposure (grey), noise alone (black), AZD5438 treatment with noise exposure (green). Mean ± SEM, P=*<0.05, **<0.01, ***<0.001 compared to noise alone by two-way ANOVA with Bonferroni post hoc test.

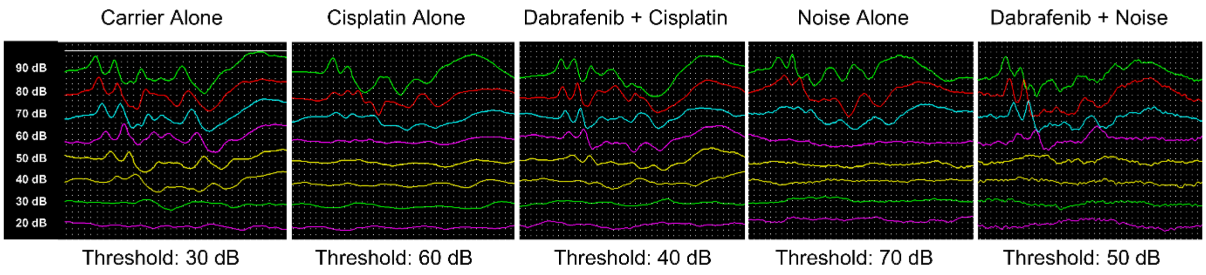

<u>Supplemental Figure 8</u> – Representative ABR trace recordings, related to Figures 4 and 5.

Representative post-ABR trace recordings from mice treated with carrier alone, cisplatin alone, dabrafenib and cisplatin cotreatment, noise exposure alone, and dabrafenib with noise exposure from experiments in Figures 4A and 5A. The threshold was recorded as the last trace at which 3 of the 5 peaks were observed.

SUPPLEMENTAL TABLES

Compound	Cisplatin Casp-3/7 IC_{50} (µM)	Cell Titer-Glo IC_{50} (µM)	Therapeutic Index
RAF-265	8.2	75	9.1
Trametinib	15	71	4.7
Vemurafenib	14	65	4.6
Dabrafenib	13	>55	>4.2
PLX-4720	38	72	1.9

Supplemental Table 1 – Table of top BRAF and MEK1/2 inhibitor hits from caspase Glo 3/7 cell-based screen, related to Figure 1.

BRAF and MEK1/2 inhibitor hits identified using a cell-based small-molecule screen in dose-response mode conducted with HEI-OC1 cell line. Compounds were screened at concentrations ranging from 1 nM to 85 µM and incubated for 22 hours. Caspase-Glo 3/7 assay (Promega) defined protective effect in the presence of 50 µM cisplatin where cells treated with cisplatin alone were assigned 100% caspase-3/7 activity and cells treated with medium alone assigned 0% caspase-3/7 activity. Hits defined as compounds that reduce caspase-3/7 activity by 50% or more in the presence of 50 µM cisplatin. Cell Titer-Glo (CTG) (Promega) defined cell viability using cells treated with compound-alone. Therapeutic index calculated as CTG IC_{50} divided by cisplatin caspase 3/7 protection IC_{50}. Top hits include BRAF inhibitors RAF-265, Vemurafenib, dabrafenib, and PLX-4720 as well as MEK1/2 inhibitor trametinib.
